# Microbiome-by-ethanol interactions impact Drosophila melanogaster fitness, physiology, and behavior

**DOI:** 10.1016/j.isci.2022.104000

**Published:** 2022-02-28

**Authors:** James Angus Chandler, Lina Victoria Innocent, Daniel Jonathan Martinez, Isaac Li Huang, Jane Lani Yang, Michael Bruce Eisen, William Basil Ludington

**Affiliations:** 1 Department of Molecular and Cell Biology, University of California, Berkeley, CA 94720, USA; 2 Department of Embryology, Carnegie Institution for Science, Baltimore, MD 21218, USA; 3 Department of Integrative Biology, University of California, Berkeley, CA 94720, USA; 4 Howard Hughes Medical Institute, Chevy Chase, MD, USA; 5 Department of Biology, Johns Hopkins University, Baltimore, MD 21218, USA

**Keywords:** Biological sciences, Genetics, Microbiology, Microbiome, Molecular biology, Physiology

## Abstract

The gut microbiota can affect how animals respond to ingested toxins, such as ethanol, which is prevalent in the diets of diverse animals and often leads to negative health outcomes in humans. Ethanol is a complex dietary factor because it acts as a toxin, behavioral manipulator, and nutritional source, with both direct effects on the host as well as indirect ones through the microbiome. Here, we developed a model for chronic, non-intoxicating ethanol ingestion in the adult fruit fly, *Drosophila melanogaster*, and paired this with the tractability of the fly gut microbiota, which can be experimentally removed. We linked numerous physiological, behavioral, and transcriptional variables to fly fitness, including a combination of intestinal barrier integrity, stored triglyceride levels, feeding behavior, and the immunodeficiency pathway. Our results reveal a complex tradeoff between lifespan and fecundity that is microbiome-dependent and modulated by dietary ethanol and feeding behavior.

## Highlights

• Flies produce more eggs on a low ethanol diet despite ingesting fewer calories
• This increased reproductive efficiency does not occur for germ-free flies
• Ethanol and commensal bacteria trigger immunity in a non-additive way
• Intestinal barrier integrity is higher in flies consuming a low ethanol diet

## Graphical abstract

**Figure:**
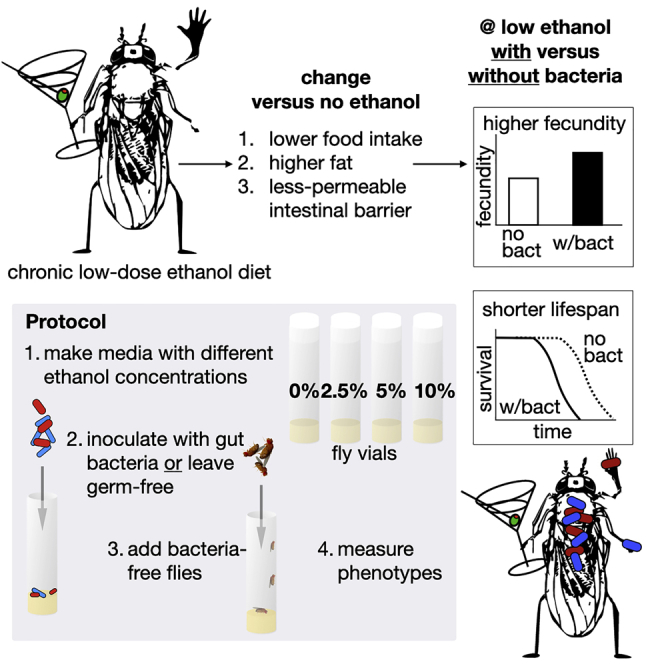

## Introduction

Microbial communities in the gut influence animal fitness (McFall-Ngai et al., 2013). They can break down dietary toxins (Kikuchi et al., 2012; Kohl et al., 2014), but they can also compromise intestinal integrity, allowing intestinal bacteria to enter the body cavity and induce sepsis (Broderick et al., 2006; Mason et al., 2011). Ethanol is a potentially toxic compound in the diets of many animals (Hockings et al., 2015; Mazeh et al., 2008; Sánchez et al., 2004; Wiens et al., 2008) and one of the most abused drugs in the world (World Health Organization, 2019). For humans, chronic alcohol consumption leads to liver failure and shortened lifespan (Gustot and Jalan, 2019). The role of microbes and microbial molecules including endotoxin (Bode and Bode, 2005), have long been implicated in alcoholic liver disease (Bhagwandeen et al., 1987; Uesugi et al., 2002). Recently, a causative role of specific gut microbiomes has been established in the susceptibility to alcohol-induced liver injury, which is transmissible from humans to mice (Llopis et al., 2016). Although associated bacterial taxa have been identified (Bluemel et al., 2020; Llopis et al., 2016), the vast diversity of the mammalian gut flora (Sender et al., 2016) complicates attribution of causation to specific bacteria (Sarin et al., 2019). Furthermore, alcohol directly increases the permeability of the intestine, which stimulates immunity at least in part by allowing microbes and their byproducts to translocate across the gut wall, where they travel to the liver and cause hepatic steatosis, inflammation, and cirrhosis (Sarin et al., 2019). Many of the negative effects of alcohol consumption can be negated by co-ingestion of probiotics, including *Lactobacillus rhamnosus* GG (Bull-Otterson et al., 2013; Forsyth et al., 2009; Li and Jasper, 2016).

A simpler model system could help dissect the complexity of host x microbiome x ethanol interactions. The common fruit fly, *Drosophila melanogaster*, is a powerful and inexpensive genetic model organism (Schneider, 2000). *D.* melanogaster is also a model for investigating the effects of ethanol on animals (Devineni and Heberlein, 2013), with a focus on larval development and adult behavior. Flies naturally consume ethanol (Devineni and Heberlein, 2009; Ja et al., 2007) and display many hallmarks of human alcoholism including tolerance, addiction, and withdrawal (Devineni and Heberlein, 2009; Ghezzi et al., 2014; Kaun et al., 2011; Robinson et al., 2012). The developmental effects of ethanol have been studied in fly larvae with a strong correspondence to human phenotypes (Logan-Garbisch et al., 2015; McClure et al., 2011). However, many of the health risks of ethanol are because of chronic rather than transient ingestion (Bluemel et al., 2020). Also, the role of long-term oral ingestion of moderate ethanol in adult flies has not been investigated, as previous studies focused on adult intoxication through ethanol vapor.

*Drosophila* is a powerful model system for investigating the commensal animal microbiome, with greatly reduced complexity compared to mammals (Broderick and Lemaitre, 2012; Douglas, 2018). Flies can be cleared of their microbial communities so that both direct and indirect effects of the microbiome can be investigated (Koyle et al., 2016). Microbiome experiments in flies can be done on a large scale, testing many variables in parallel (Wong et al., 2014). Studies have cataloged the microbiome of flies, demonstrating an overlap in taxonomy with humans, with Firmicutes (primarily *Lactobacilli*) and Proteobacteria (primarily *Acetobacter*) making up the majority of the taxa (Broderick and Lemaitre, 2012; Chandler et al., 2011; Wong et al., 2013). Furthermore, these commensal bacteria affect many components of fly fitness and physiology (Shin et al., 2011; Storelli et al., 2011; Wong et al., 2014).

Here we used flies to deconstruct the complex interplay between host, microbiome, and ingested ethanol. We (1) developed a model of chronic alcohol consumption, (2) quantified the effects on both microbiome and host, and (3) differentiated between behavioral and physiological causes of the effects. We find that fly fitness in the presence of ethanol is influenced by the microbiome as well as feeding, intestinal permeability, lipid content, and the immune response. Taken together, our model provides a baseline by which to investigate the complex effects of alcohol and bacteria on animal physiology, including toxic effects, behavioral modification, and nutrition.

## Results and discussion

### Establishing a model for chronic ethanol ingestion in flies

Previous studies of ethanol in adult flies used ethanol vapor, likely bypassing the gut microbiome. Here, we administered ethanol directly in the food. To measure the effect of the microbiome, we used two microbiome treatments: bacteria-free and bacterially-colonized (a.k.a. conventionally-reared). Bacteria-free flies were generated using established protocols (Koyle et al., 2016) and bacterially-colonized flies were created by allowing approximately 50 conventionally-raised adults (from unmanipulated lab stocks) to seed autoclaved media with their frass, removing these flies, and then introducing bacteria-free flies.

Except as noted otherwise, we focused our assays on mated female flies that were 5–7 days after eclosion because the microbiome differs by sex (Han et al., 2017) and mating status (Lee et al., 2013b); gut development is not complete until approximately two days after eclosion (O’Brien et al., 2011); the gut undergoes significant expansion after mating (Miguel-Aliaga et al., 2018); and colonization of the gut is not significant until 4 days after eclosion, potentially reflecting additional developmental processes (Blum et al., 2013). Furthermore, ethanol intake is mating-dependent in male flies (Shohat-Ophir et al., 2012), potentially adding variation to assays using males.

**Figure 1:**
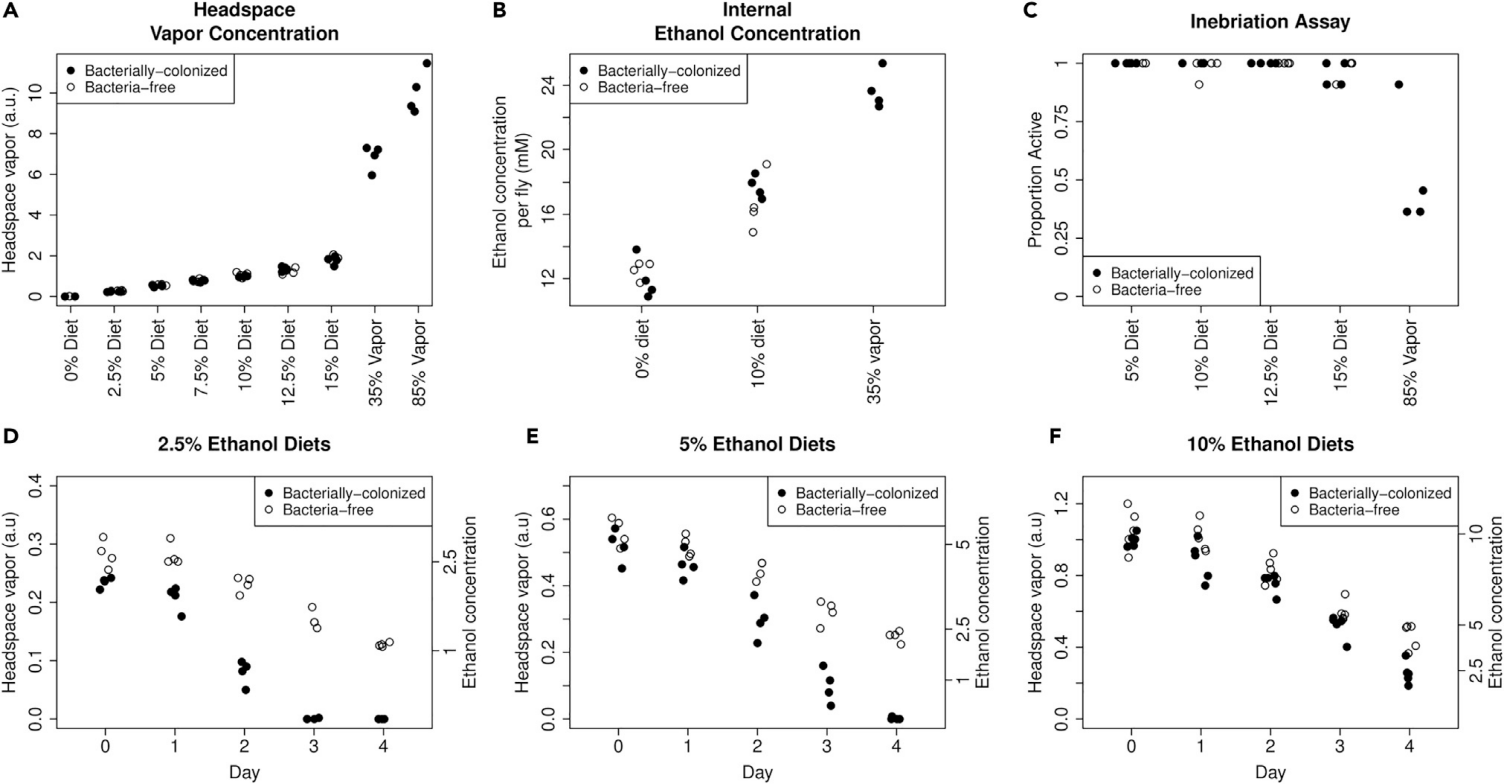
Establishing a chronic ethanol ingestion model in *Drosophila* (A) Dietary ethanol produced lower headspace ethanol vapor than standard methods for fly ethanol exposure. Headspace vapor was determined by sampling the vial headspace with a syringe and forcing this mixture through a medical-grade breathalyzer. For the 35 and 85% ethanol treatments, either a cotton ball (35%) or a cellulose acetate plug (85%) was soaked with liquid ethanol in a tightly capped vial. (B) Flies up took ethanol from their diet, which produced lower internal ethanol concentrations than 35% ethanol vapor. Internal ethanol concentration was assayed enzymatically on individual flies fed either 0% or 10% ethanol diets or exposed to 35% ethanol vapor. (C) Dietary ethanol did not cause inebriation by a standard assay based on activity. Proportion active was the proportion of 11 individual flies that stand up after gently tapping the vial 30 min after initial exposure to ethanol. 85% ethanol vapor (final column) led to inebriation in about half of the individuals at 30 min. (D–F) Dietary ethanol content decreased over time with greater loss in bacterially-colonized treatments. Ethanol concentration (right axis) was calculated from day 0 measurements (Figure S1). Measurements from 0% ethanol media were always below 0.02 and therefore not shown. See also Figure S1.

We measured the ethanol in our experiments by using a commercial breathalyzer to sample the headspace vapor of freshly prepared vials with 0%–15% dietary ethanol. For comparison, we used two methods to expose flies to ethanol vapor. First, we soaked a cotton ball with 2 mL of 35% ethanol and covered the wet cotton ball with a dry one so flies could not ingest the ethanol [similar to inebriation studies, e.g. (Fry, 2014)]. Second, we added 1 mL of 85% ethanol to a cellulose acetate plug. Headspace vapor precisely measured dietary ethanol (Figures 1A and S1) [following (Morton et al., 2014)]. We also found that the established ethanol vapor administration methods led to ethanol vapor levels many times greater than our dietary ethanol method (Figure 1A). Therefore, our chronic ingestion model exposed flies to a much lower headspace vapor than previously established acute inebriation models.

To confirm that flies effectively uptake ethanol, we measured the internal ethanol concentrations of flies. We found that flies fed 10% ethanol diets contained higher internal concentrations of ethanol than flies fed 0% ethanol diets, which shows that in our treatment conditions flies successfully ingested dietary ethanol (Figure 1B). Flies exposed to 35% ethanol vapor had even higher internal ethanol levels (Figure 1B), indicating that our dietary regime exposes flies to lower levels of ethanol. Notably, bacterially-colonized and bacteria-free flies on 10% ethanol food had the same internal ethanol concentration, indicating bacteria do not rapidly degrade the ethanol.

We tested for inebriation with a standard assay, which measures the inability of flies to stand after gently tapping the vials (Sandhu et al., 2015). After 30 min, ∼5% of flies on a 15% diet showed signs of inebriation, whereas almost no inebriation occurred with lower ethanol diets (Figure 1C). In contrast, ∼50% of flies exposed to 85% ethanol vapor were inebriated. Bacterial treatment did not affect inebriation (Figure 1C).

Finally, we asked to what extent evaporation and bacterial metabolism reduce ethanol content. Ethanol vapor decreased over time (Figures 1D and 1E). With 10% ethanol media, approximately half the ethanol remained after 3 days. At lower doses of ethanol, bacteria increased the ethanol loss rate. 5% ethanol vials with bacteria-colonized flies had ethanol levels ∼50% of bacteria-free flies at the 3-day mark, whereas in the 10% ethanol treatment no difference in the ethanol concentrations between bacterially-colonized and uncolonized flies was apparent until day four. These results suggest ethanol loss by two mechanisms. First, evaporation decreased ethanol concentration. Second, bacterial metabolism consumed ethanol. The lag in bacterial ethanol degradation may occur because initial bacterial populations in the vials are small, and high initial ethanol concentrations may inhibit bacterial metabolism. In addition, bacterial influence on the host could remove ethanol, for example, by larger larvae consuming more ethanol or simply churning the food to cause more off gassing of ethanol. Overall, our method establishes an experimental model of chronic ethanol ingestion in adult *D. melanogaster*. Ethanol remains in the media long enough for flies to uptake it, ethanol is detectable internally after ingestion, and flies do not show overt inebriation as assessed in the climbing assay.

### Bacterial colonization of flies masks the negative effects of ethanol on lifespan

We measured the combined effects of ethanol and the microbiome on fly lifespan and fecundity, which have not been investigated in the context of a fly ethanol model. In general, ethanol consumption in humans is associated with shorter lifespan (Wood et al., 2018). The natural habitat of *D. melanogaster*, fermenting fruit, often contains 1–5% ethanol and can be as high as 10% ethanol where flies are present in vineyards (Gibson et al., 1981). We first examined flies at 0% and 5% ethanol in flies with or without bacteria. We measured lifespan, fecundity, and microbiome composition (see next section) in the same experiment. In this experiment, we transferred flies to fresh food every 3–4 days to balance between maintaining dietary ethanol concentration, which decreased over time, (cf. Figures 1D–1F) and maintaining bacterial colonization, which has been shown to be more stable for lab fly bacteria when less frequent transfers are made (Blum et al., 2013).

**Figure 2:**
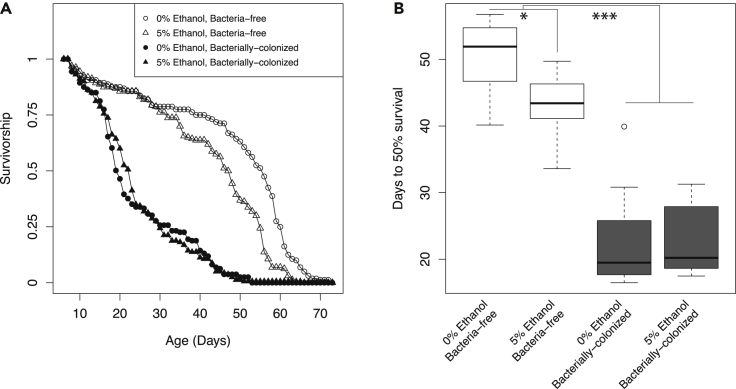
Bacteria mediate the effect of ethanol on fly lifespan (A and B) Survivorship and (B) days to 50% survival of bacterially-colonized and bacteria-free flies fed diets containing 0% or 5% ethanol. Bacterial colonization and ethanol reduced fly lifespan, with the negative effect of ethanol greater in magnitude and more significant in bacteria-free flies. Survival is time from adult eclosion from the pupal case (see STAR Methods) and data from individual flies was pooled from two biological replicates of 4 vials each. 120 flies total per treatment. Days to 50% survival is per replicate vial. In (B), box indicates quartiles, line indicates median, whiskers are 1.5 times the interquartile range. Outlier is an open dot. Statistical test was an ANOVA followed by post hoc comparison between 0% and 5% ethanol in germfree flies (p = 0.014, ∗) and bacteria-free versus bacterially colonized (p < 0.001, ∗∗∗) by Welch’s *t*-test. See also Figures S2–S4, and S11.

Bacterially-colonized flies consistently showed a shorter lifespan than bacteria-free flies (Figures 2A and 2B), in agreement with previous studies from our lab (Figures S2 and S3 and Table S1) and others (Clark et al., 2015; Lee et al., 2019; Ridley et al., 2012; Steinfeld, 1927). We note that our data are reasonable with respect to other studies as mean fly lifespans vary from roughly 20 to 60 days depending on fly genetic background and dietary nutrition (Keebaugh et al., 2019; Lesperance and Broderick, 2020) and that ethanol likely shortens lifespan through its role as a toxin (De Nobrega et al., 2017).

To probe the effects of ethanol dose, we next repeated the experiment with seven gradations of ethanol. At higher than 5% ethanol, there was no difference in lifespan between bacterially-colonized flies and bacteria-free. Bacteria-free flies, which overall lived longer, showed a nearly linear and dose-dependent decrease in average and maximum lifespan beginning at just 2.5% ethanol (Figure S4). These data were consistent with the original experiment done on only 0% and 5% ethanol (Figure S3).

Overall, these data indicate that bacterial colonization and ethanol exposure both limit lifespan. Negative effects of bacterial colonization on lifespan have consistently been observed in flies fed rich diets, but not flies on nutrient-poor diet (Keebaugh et al., 2019). Ethanol reduced lifespan in bacteria-free flies, but the bacterial effect was dominant to the ethanol effect at low to moderate ethanol, indicating a bacteria by ethanol interaction influencing fly lifespan (Robust 2-way ANOVA: p < 0.001, Figure S4).

### Bacteria ameliorate the negative effects of ethanol on fecundity

**Figure 3:**
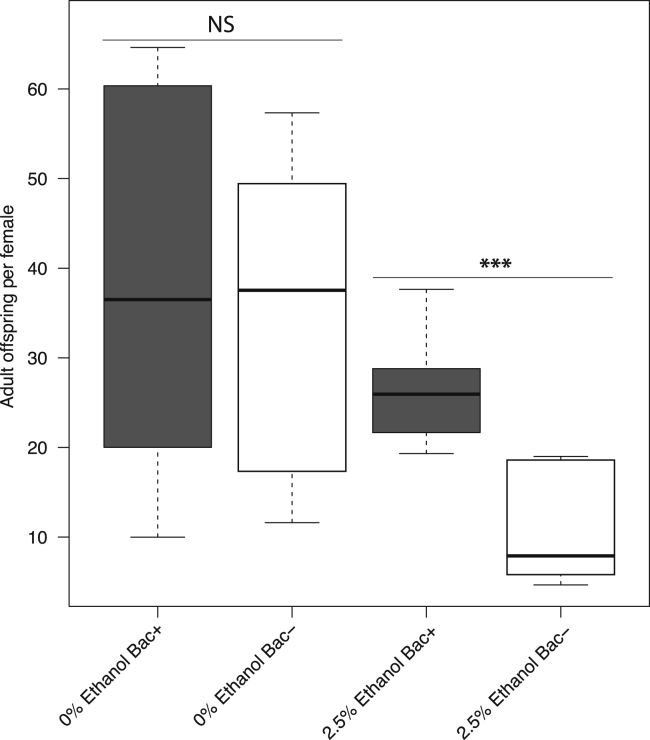
Bacteria mediate the effect of ethanol on fly fecundity Adults per female was calculated as the average total number of progeny per female over the entire lifetime (see STAR Methods). Box indicates quartiles, line indicates median, whiskers are 1.5 times the interquartile range. 8 replicates per treatment set up on two separate dates. 120 female parents per treatment. p-values are calculated from a pairwise *t*-test between bacterial treatments for a given ethanol concentration and are Bonferroni corrected for multiple comparisons (p < 0.001, ∗∗∗). See also Figures S5–S7, and S11.

Ethanol reduced fly fecundity, with bacteria reducing the severity of the effect (Figures 3 and S5). Without ethanol, we observed no difference in fecundity between bacteria-colonized and bacteria-free flies, consistent with previous work (Ridley et al., 2012). Fecundity of bacteria-free flies was more reduced at 2.5% ethanol compared with bacteria-colonized flies (pairwise *t*-test: p < 0.001, Figure 3). To determine whether this trend was apparent at higher ethanol concentrations, we measured the number of adult progeny produced per female over their lifespan over a range of ethanol concentrations from 0 to 15%. Higher fecundity was observed in bacterially-colonized flies at both 5% and 7.5% ethanol diets, though these were not individually significant at p < 0.05 after Bonferroni correction for multiple comparisons (Figure S5A). At higher ethanol concentrations, fecundity was extremely limited regardless of bacterial treatment.

We next sought to address several caveats of the experimental setup, which was optimized for measuring lifespan and fecundity concurrently. On 2.5% ethanol media, ethanol content was significantly reduced by day two in the bacterially-colonized treatment (Figure 1D), meaning the difference in fecundity observed in the 2.5% ethanol treatment (Figure 3) could simply be because of less dietary ethanol in the bacterially-colonized treatment. To test this, we first accounted for the loss statistically, finding that the magnitude of the ethanol loss is not proportional to the fecundity increase (Data S1). We next experimentally tested the effect of ethanol loss by repeating the fecundity experiment but transferring the flies to fresh diets every day, which minimizes ethanol loss. To ensure the persistence of the intestinal bacterial communities, we seeded new media with the frass of bacterially-colonized flies as before. Bacterially-colonized flies had greater fecundity in the 2.5% ethanol treatment relative to bacteria-free flies in the daily transfer experiment (pairwise *t*-test: p = 1 × 10^−5^, Figure S5B), indicating that ethanol loss does not account for the increased fecundity of bacterially colonized flies.

We also reasoned that calories from ethanol might spur the bacteria to grow more and thus allow the flies to acquire more nutrition from the food. Germ free flies would not have the bacteria to convert the ethanol from toxin to nutrition and thus would not receive this benefit. As a test of whether calories alone could aid fecundity, we added the amount of glucose that makes up the difference in calories between the 0% and 2.5% ethanol diets. We assayed this isocaloric diet concurrently with the 1 day transfer experiments.

We found no effect of bacterial treatment in the isocaloric diets (Figure S5B) suggesting that the differences in fecundity are not due simply to ethanol’s caloric contribution but rather are consistent with its role as a toxin. This result does not rule out that other sources of calories, such as protein or fat, could have different effects, but we leave that line of inquiry to future studies.

Effects on fecundity could be due either to larval survival or maternal egg laying, and we designed experiments to differentiate these. We could not directly count egg laying in the experiments where flies were transferred to new media every 3–4 days, and thus could not calculate survival from egg to adulthood. As a proxy, we measured time to first pupa formation and the percentage of flies with successful eclosion from the pupal case. Development time increased with increasing ethanol (Figure S6A), supporting previous observations that ethanol slows development (McClure et al., 2011). However, we found no differential effect on development time between bacterially-colonized and bacteria-free treatments at the 0%, and 2.5% ethanol treatments (all pairwise *t*-tests, p > 0.2), indicating that development time does not account for the observed fecundity differences in these treatments. Our results are consistent with a previous study showing that ethanol does not affect larval development except in the final larval stage at 5 days (McClure et al., 2011), when most of the ethanol has evaporated from the media (Figures 1D–1F). Reduced eclosion at higher ethanol was not sufficient to account for the major differences in fecundity observed nor the differential effect on development time between bacterially-colonized and bacteria-free treatments at the 0%, and 2.5% ethanol treatments (Figure S6B).

The other major variable in fecundity is egg laying. To test whether egg laying can explain the fecundity results, we reared flies on their ethanol media for 5 days and then transferred individual females to vials with a single male and sterile molasses media with fresh, wet yeast paste, which stimulates laying of any eggs that are already mature in the female. Flies were removed after a 24 h period and the eggs manually counted. Because it takes ∼24 h to produce new, mature eggs, the vast majority of eggs that we counted were produced when the flies were on the ethanol food. Thus, this method measures the fly’s egg-laying capacity on the ethanol media rather than a behavioral choice. Bacteria-free flies laid fewer eggs on both 0% and 2.5% ethanol diets, with a non-significant difference on 5% ethanol (Figure S7). This trend is consistent with the results in Figures 3 and S5. Taken together, our results suggest that maternal egg production strongly contributes to a differential increase in fly fecundity in bacterially colonized flies on a 2.5% ethanol diet.

### A tradeoff between lifespan and fecundity is apparent for some ethanol concentrations

**Figure 4:**
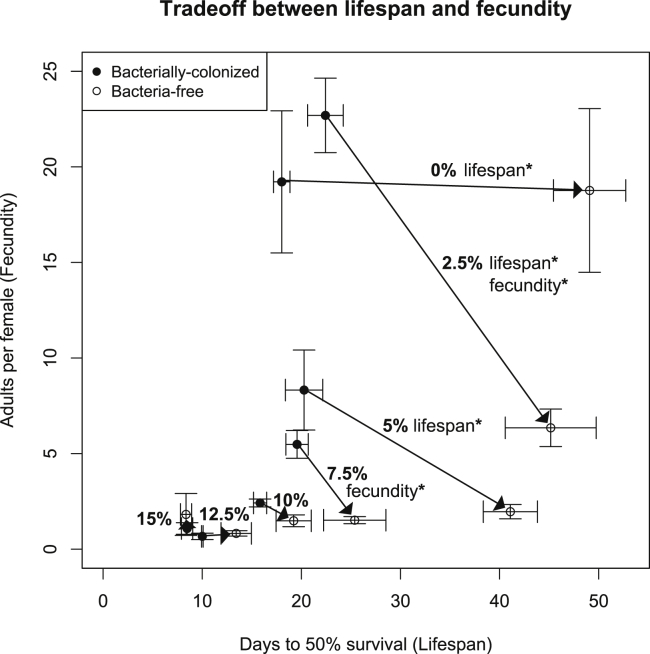
A tradeoff between lifespan and fecundity is apparent for some ethanol concentrations X-axis: Mean days to 50% survival (from Figure 2). Y-axis: Mean adult progeny per female (from Figure 3). The number on the arrow indicates the dietary ethanol concentration. A tradeoff between lifespan and fecundity based on bacterial presence/absence is apparent at individual ethanol concentrations of 2.5%, 5%, and 7.5% but is only significant for both lifespan and fecundity at 2.5%. Data presented as mean ± SEM between replicate vials. Results are from a single biological replicate with 4 experimental replicates per ethanol concentration and 1,120 adult flies total at the start of the experiment. Significance is for Bonferroni-corrected alpha (p < 0.05, ∗).

We and others have previously observed that the microbiome can produce indirect life history tradeoffs between lifespan and fecundity (Gould et al., 2018; Walters et al., 2020). In this tradeoff, an increase in fecundity is correlated but not causative of a shorter lifespan. The decrease in lifespan has been suggested to stem from an increase in calories, i.e. less dietary restriction (Barnes et al., 2008; Keebaugh et al., 2019). We plotted the data from the experiment where lifespan and fecundity were measured concurrently over a range from 0% to 15% ethanol (Figure 4). In contrast to the previous reports, we found that ethanol does not lead to a typical tradeoff between lifespan and fecundity observed by varying nutrients (Djawdan et al., 1996; Zera and Harshman, 2001), rather at each ethanol concentration, we observed higher fecundity and shorter lifespan in flies with bacteria than without, but this tradeoff was only significant at 2.5% ethanol, which is similar to natural fly habitat. At all other concentrations, ethanol decreased both components of fitness (Figure 4), suggesting that ethanol, even at the low concentrations used in this study, acts more like a toxin than a source of calories.

### Ethanol shifts the composition of bacteria associated with D. melanogaster

Diet is a strong determinant of microbiome composition in flies and other animals (David et al., 2013). In particular, fruit feeding flies, which are exposed to naturally produced dietary ethanol, have significantly different bacterial and yeast communities than flies collected from other substrates (Chandler et al., 2011, 2012). We hypothesized that the bacterial communities associated with flies would shift in response to ethanol ingestion. In particular, we expected that ethanol would decrease the total abundance of bacteria in high ethanol treatments and these shifts would favor the abundance of bacteria with low sensitivity to ethanol. Thus, in a parallel replicate of the lifespan-fecundity experiment (c.f. Figures 3, 4, S4, and S5), we determined fly bacterial load and composition by homogenizing individual flies and plating onto selective media. The different bacterial strains were identified by colony morphology, which we previously evaluated by deep sequencing 16S rRNA amplicon libraries from conventionally-raised flies (Aranda-Díaz et al., 2020) and confirmed here by 16S rRNA Sanger sequencing of colonies.

**Figure 5:**
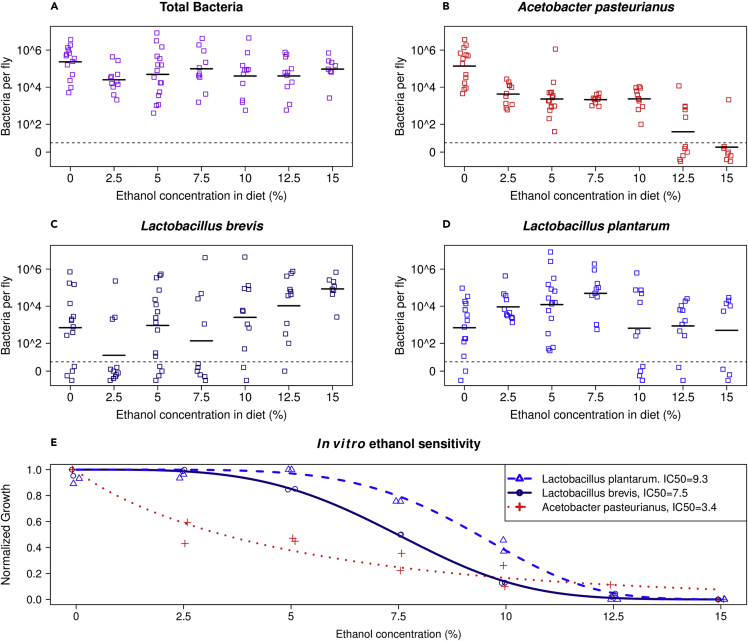
Bacterial community dynamics in response to ethanol diets (A–D) Total CFUs, (B) *A. pasteurianus* CFUs, (C) *L. brevis* CFUs, and (D) *L. plantarum* CFUs. The abundance of *Acetobacter* decreased with increasing dietary ethanol, while the abundance of *Lactobacillus plantarum* decreased slightly and *Lactobacillus brevis* increased. Each point represents an individual fly. All points below the dashed line are 0 CFUs and are jittered for clarity. The black bars represent the mean of the log transformed bacterial load. Number of individual flies per ethanol treatment was: 0% N = 14; 2.5% N = 11; 5% N = 16; 7.5% N = 10; 10% N = 11; 12.5% N = 11; 15% N = 8 from two separate biological replicates. There was no effect of fly age on bacterial composition [multivariate ANOVA (Adonis, package vegan in R; p = 0.159)] and therefore all four timepoints were pooled (see STAR Methods). (E) *In vitro*, *Acetobacter* is more sensitive to ethanol than *L. plantarum* or *L. brevis*. Growth was measured using MRS (*Lactobacilli*) or MYPL (*Acetobacter*) liquid media containing 0%–15% ethanol in a 96-well plate for 24 h with shaking at 30°C. 12 technical replicates of each strain at each ethanol concentration per plate. Data points are the final normalized OD of two independent biological replicates. A two-parameter Weibull function was fit to the normalized ODs from the aggregate data for each strain (R package drc: Analysis of Dose-Response Curves). The inhibitory concentration for 50% growth (IC50) was calculated as the ethanol percentage that reduced normalized maximum OD by half. See also Figure S8.

We found that total bacterial load per fly was between 9 × 10^3^ and 3 × 10^6^ colony forming units (CFUs) for the 0% ethanol containing diets (mean = 7 × 10^5^ CFU). This is comparable to previous studies of *D. melanogaster* (Blum et al., 2013; Obadia et al., 2017). Contrary to our expectations that ethanol would decrease bacterial load, we found that the mean total bacterial load was relatively constant up through 15% dietary ethanol (Figure 5A). However, the abundance of the different bacteria types changed in response to ethanol concentration. *Acetobacter* abundances decreased 10-fold from 0% to 2.5% ethanol and remained constant up to 12.5% ethanol where they dropped to essentially 0 (Figure 5B). Conversely, we found that the response of the *Lactobacilli* to ethanol was remarkably different from *Acetobacter*. The abundance of *Lactobacillus brevis* increased with dietary ethanol and this was the only species that was present in all flies at 15% ethanol (Figure 5C). *Lactobacillus plantarum* was most abundant at intermediate concentrations of ethanol, but like *L. brevis*, it did not appear as sensitive to ethanol as *Acetobacter* (Figure 5D).

To separately measure the effect of ethanol on bacterial growth, we measured *in vitro* growth of *Acetobacter*, *L. plantarum*, and *L. brevis* strains isolated during the experiment in Figures 5A–5D. These experiments confirmed that *Acetobacter* was more sensitive to ethanol than *L. brevis* and *L. plantarum* (Figure 5E). Acetobacters are known ethanol degraders (Shimizu et al., 1977), which might be reflected in their lower tolerance since they decrease ethanol concentrations and prevent them from reaching toxic levels. Lactobacilli are known wine souring organisms (Groenewald et al., 2006), which might be reflected in their ability to tolerate higher ethanol concentrations. These results indicate that the bacterial composition of flies varies, at least in part, according to the ethanol sensitivities of the bacterial strains. However, *L. brevis* has a lower tolerance than *L. plantarum in vitro* but a higher abundance *in vivo*. Noting that *L. brevis* is often associated with more pathological effects on flies (Ferguson et al., 2021; Lee et al., 2013a), we speculate that the discrepancy between ethanol tolerance *in vitro* and abundance in the flies involves the effects of ethanol on the physiology of flies. Overall, the bacteria appear more sensitive to ethanol *in vitro* than *in vivo*. We speculate that the fly gut environment protects bacteria from ethanol toxicity. In support of this, we found a high abundance of *L. brevis* and *L. plantarum* within flies fed a 15% ethanol diet despite these bacteria being undetectable on the 15% ethanol fly food (Figure S8). Similarly, *Acetobacter* is present within flies fed 12.5% ethanol despite this bacterium being absent on the fly food at 12.5% ethanol (Figure S8), which could have implications for detecting *Acetobacter* in wild fly habitat, such as fortified wine production facilities (Gibson et al., 1981). These results are consistent with the host shielding gut bacteria from the effect of ethanol.

### Dietary ethanol increases intestinal barrier integrity

**Figure 6:**
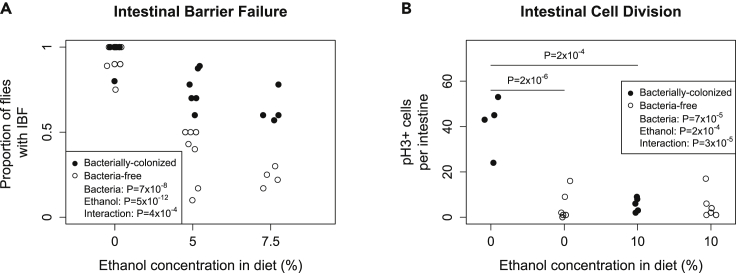
Bacteria mediate some effects of ethanol on fly physiology (A) The prevalence of intestinal barrier failure (IBF) decreased with dietary ethanol and this decrease was greater in bacteria-free flies. Each point represents the average from a replicate vial. Flies were scored within 24 h before death. Females only in this panel. Males show the same trend (Figure S9A). (B) Ethanol reduced the number of mitotic cells in the intestine in bacterially-colonized, but not bacteria-free flies, which had a low mitotic index regardless of ethanol treatment. Each data point indicates the number of anti-PH3+ stained cells in an individual intestine. Within each panel, values in the legend are the results either from an ANCOVA (A) or a two-way ANOVA(B). Comparison p-values were calculated from a pairwise *t*-test between treatments and were Holm-Bonferroni corrected for multiple comparisons within an experiment. See also Figure S9.

To explore the causes of the bacterially-mediated responses to ethanol, we investigated numerous physiological, behavioral, and transcriptional phenotypes. We first examined intestinal barrier failure (IBF), which is strongly linked to alcoholic liver disease in humans (Chen and Schnabl, 2014) and is a hallmark of aging-related death in flies (Clark et al., 2015; Rera et al., 2012). We used the Smurf assay (Rera et al., 2012), rearing flies on a diet containing blue dye no. 1 and scoring them for a blue body coloration, which is indicative of a permeabilized gut. Consistent with previous results (Clark et al., 2015; Rera et al., 2012), we found that nearly all flies on a 0% ethanol diet showed IBF upon death. Surprisingly, on ethanol diets, we found a significant decrease in the proportion of flies with IBF (Figures 6A and S9A). Furthermore, bacteria-free flies (which were more sensitive to ethanol) showed significantly less IBF than bacterially-colonized flies on ethanol diets, indicating that IBF is not the primary cause of death on an ethanol diet, in contrast to a diet without ethanol. Bacteria and ethanol showed a significant statistical interaction in this assay (ANOVA: p < 0.001, Figures 6A and S9A), suggesting the response to bacteria may be changed on an ethanol diet.

We note that this result does not agree with some literature from humans and other mammals. One study utilizing bacteria-free mice found that gut permeability only ensues after ethanol consumption when normal gut microbiota are present (Canesso et al., 2014). However, that study used an acute binge of alcohol ingestion, which disrupts the intestinal mucous membrane (Bode and Bode, 2005). By supplying chronic low doses of ethanol as part of the diet, we circumvented the direct chemical effect of alcohol on mucous membranes. Thus, we cannot directly compare the results.

### The effect of ethanol on intestinal cell division is microbiome dependent

Intestinal barrier function is maintained through controlled intestinal stem cell (ISC) turnover (Lemaitre and Miguel-Aliaga, 2013). Because stem cell hyper-proliferation leads to loss of intestinal function (Li and Jasper, 2016), we hypothesized that the reduction in IBF was due to a decrease in ISC turnover. As a proxy for stem cell turnover, we measured mitotic cells in the gut by phospho-histone H3 (PH3) antibody staining (Apidianakis and Rahme, 2011). In two independent experiments, we found that in the absence of ethanol, there were significantly more mitotic cells in bacterially-colonized flies (pairwise *t*-test: p = 2 × 10^−6^, Figure 6B; pairwise *t*-test: p = 2 × 10^−6^, Figure S10), consistent with previous work of other labs (Buchon et al., 2009a; Guo et al., 2014).

In the presence of ethanol, we found that baseline ISC division was significantly decreased for bacterially-colonized flies, but unchanged in bacteria-free flies (pairwise *t*-test: p = 2 × 10^−4^, Figure 6B; pairwise *t*-test: p = 3 × 10^−6^, Figure S10). These results are in accord with the IBF data presented in Figure 6A for bacterially-colonized flies, and suggest that there is less intestinal damage in flies fed ethanol.

The decrease in mitotic cells in bacterially-colonized flies led us to hypothesize that ethanol might inhibit intestinal regeneration following a biological or chemical challenge. To test this hypothesis, we infected flies with *Erwinia carotovora carotovora 15* (Ecc15), a non-lethal pathogen of *Drosophila*, which induces mitotic divisions following oral ingestion (Buchon et al., 2009b). In both bacteria-free and bacterially-colonized flies ingesting 10% ethanol, infection with Ecc15 increased the number of PH3 positive cells (pairwise *t*-tests: p = 0.021 and p = 0.013, respectively, Figure S9B). Thus, ethanol does not inhibit the regenerative capacity of the gut suggesting ethanol reduced baseline levels of cell division rather than the response to intestinal damage. Based on the reduction of intestinal barrier failure (Figures 6A and S9A), the reduction of PH3 positive cells (Figure 6B), and the normal response to pathogen ingestion (Figure S9B), ethanol-induced mortality does not appear to occur through diminished intestinal integrity.

### Ethanol ingestion increases stored triglycerides in flies independent of the microbiome

The maintenance of intestinal homeostasis with ethanol treatment may be because of a change in overall fly metabolism. In flies, poor quality diets are linked to both ISC turnover and obesity (Regan et al., 2016; Skorupa et al., 2008). In humans, increased fat deposits in the liver are a hallmark of alcoholic liver disease (Diehl, 2002). We hypothesized that ethanol ingestion might lead to greater accumulation of stored triglycerides in flies. Triglycerides are a primary molecule for fat storage in flies and are mainly found in adipocytes within the fat body, an organ analogous to the mammalian liver that is responsible for the majority of energy reserves in adult flies (Arrese and Soulages, 2010). We therefore measured stored triglycerides in bacteria-free and bacterially-colonized flies on 0%, 5% or 10% ethanol diets. Consistent with our hypothesis, we found that dietary ethanol increased triglycerides regardless of bacterial colonization, with no effect on either total fly mass or free glycerides (Figures S9C and S9D and Table S2). Thus, ethanol-induced fat accumulation does not depend on intestinal barrier disruption, which is greater in bacterially-colonized flies (Figures 6A and S9A). It is notable that the change in fat content does not correspond to a change in overall mass. This supports the hypothesis that ethanol changes the fly’s overall metabolism.

### Ethanol ingestion and bacterial colonization affect expression of innate immunity genes

The fat body is a major immune organ in flies, producing many of the antimicrobial peptides (AMPs) that are a major class of immune effector in flies and mammals. AMPs production has been shown to shorten lifespan in flies (Hanson et al., 2019). Innate immunity has also been implicated in alcoholic fatty liver disease in mammals. We hypothesized that ethanol increases baseline immunity. To test, we developed a custom NanoStrings probeset with selected candidate genes likely to be influenced by ethanol or the microbiome. In concordance with previous work (Broderick et al., 2014), we found immunity (e.g. lysozyme X and the PGRPs), stress response (e.g. GstD5 and HSP23), and cell differentiation-induction (e.g. upd3) genes to be upregulated in response to bacterial colonization (Table 1). Likewise, and in agreement with (Broderick et al., 2014; Elya et al., 2016), we found that antimicrobial peptides (AMPs) as a group showed increased expression in bacterially-colonized treatments (Table S3).

**Table 1:** Genes showing significant expression changes in response to either ethanol ingestion or bacterial colonization

Gene	p-value	Fold-Change
**Genes affected by ethanol ingestion**		

neuropeptideF	0.015	1.5
Acetaldehyde Dehydrogenase	0.035	1.6
Acetyl-CoA synthetase	0.038	2.8
HSP70Bc	0.047	3.1
Alcohol Dehydrogenase	0.047	1.8

**Genes affected by bacterial colonization**		

PGRP SC1A/B	0.0029	3.3
upd3	0.030	5.5
LysozymeX	0.030	18
upd2	0.030	69
Crys	0.030	9
Defensin	0.030	5.3
Charon	0.030	3.8
GstD5	0.030	2.7
PGRP SD	0.030	4.2
PGRP LB	0.030	2.2
Drosomycin-like 1	0.031	44
HSP23	0.040	2.3
PGRP LC	0.048	2.7

p-values are adjusted using a 5% Benjamini-Hochberg false discovery rate correction. Genes with less than a 1.5 fold-change or with average normalized counts within two standard deviations of the negative control probes are excluded. See also Table S3.

We examined many genes and molecular pathways known to mediate the effects of ethanol intoxication in flies. Ethanol subtly yet significantly changed the expression of several canonical alcohol metabolism genes, such as alcohol dehydrogenase (Table 1). Neuropeptide F was upregulated 1.5-fold, consistent with the low observed internal ethanol concentration (Figure 1B).

**Figure 7:**
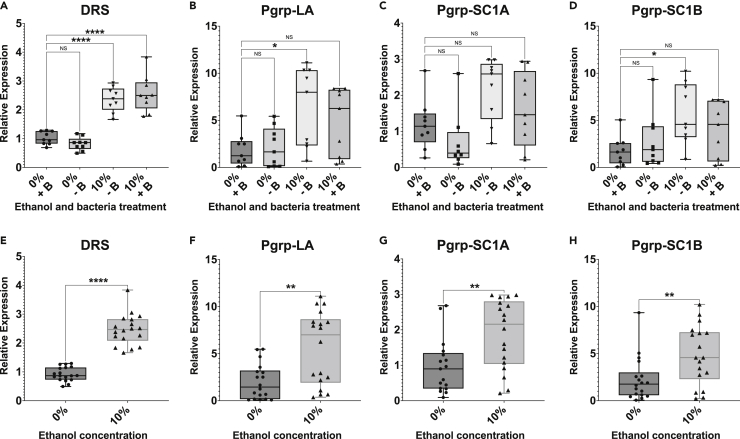
Ethanol changes the immune response (A–D) RT-qPCR of (A) Drosomycin, (B) peptidoglycan receptor protein (PGRP) LA, (C) PGRP-SC1A, and (D) PGRP-SC1B. Three biological replicates with three experimental vials each. 6 flies homogenized per replicate. Individual replicates shown. Box shows quartiles; bar is median. Relative-fold expression calculated relative to 0% ethanol bacterially-colonized fly treatment and normalized to ribosomal protein L32 (rpl32). ANOVA with multiple comparisons corrected by Bonferroni method. (E–H) Treatments were pooled by ethanol concentration to test whether ethanol increases the immune response. Corrected p-values indicated as p < 0.05, ∗; p < 0.01, ∗∗; p < 0.001, ∗∗∗; p < 0.0001. ∗∗∗∗. See also Figure S10.

We next examined whether ethanol may elicit the innate immune response. Immune activation causes shorter lifespan in flies (DeVeale et al., 2004; Eleftherianos and Castillo, 2012; Garschall and Flatt, 2019) and resistance to ethanol vapor in flies is linked to the innate immunity response (Troutwine et al., 2016). Furthermore, the innate immune response is linked to fat metabolism in flies (Davoodi et al., 2019; Martínez et al., 2020; Molaei et al., 2019), and we noted that ethanol causes an increase in TAG without an increase in weight (Figures S9C and S9D and Table S2). In mammals, dysregulation of the immune response and persistent inflammation is linked to alcoholism (Bode and Bode, 2005; Sarin et al., 2019) and aging (Gomez et al., 2008; Shaw et al., 2010). In agreement with the hypothesis, several innate immunity genes showed a change in expression on ethanol (Table S3). To confirm the NanoStrings findings, we repeated the ethanol and bacteria treatments and performed qRT-PCR, comparing three ethanol-responsive genes and six antimicrobial peptides (AMPs), which are innate immune effectors. We found broad agreement between the different gene expression techniques, with ethanol increasing expression for four of six AMPs (Figure 7) and 2 of 3 ethanol-responsive genes (Figure S10). We note that expression was increased both for AMPs that are canonically regulated by the immune deficiency pathway, which is the primary gut immunity pathway, as well as the Toll pathway, which is primarily a systemic response coordinated in the fat body (De Gregorio et al., 2002), consistent with ethanol affecting multiple pathways in fly physiology.

### Ethanol diets decrease food consumption

Food consumption can have a major effect on nearly all aspects of fly biology and is directly related to lifespan through caloric restriction (Skorupa et al., 2008). Furthermore, ethanol is known to affect food consumption (Ja et al., 2007; Park et al., 2018; Park and Ja, 2020). We therefore measured food consumption in bacterially-colonized and bacteria-free flies on diets of 0%, 2.5% and 5% ethanol using 32P-labeled dCTP incorporation (Deshpande et al., 2014). We found microbiome status made no difference in food consumption (Figure S11), which indicates that differences in lifespan and fecundity between bacterially-colonized and bacteria-free flies at a given ethanol concentration cannot be attributed to food consumption and associated caloric intake.

We did, however, find an overall decrease in food consumption because of ethanol, with flies consuming roughly half as much food for each 2.5% increase in ethanol (Figure S11) at 24 h, consistent with previous work (Ja et al., 2007; Park and Ja, 2020). The same trend with decreased magnitude was observed at 48 ha and 72 h, consistent with the loss of ethanol due to evaporation (Figures 1D–1F). Our finding that dietary ethanol decreases food consumption is critical to interpreting the fitness and physiological results presented earlier. Specifically, it means that flies on ethanol diets are ingesting substantially fewer calories: when accounting for the increased caloric value of ethanol supplemented diets, flies on 2.5% and 5% ethanol food were ingesting just 60% and 38% of the calories of 0% ethanol diets, respectively (Figure S11). Thus, the decrease in lifespan on an ethanol diet (Figure 2) occurs in spite of lower caloric intake, underscoring the role of ethanol as a toxin, even in *D. melanogaster*, which is an ethanol-adapted species.

With respect to fecundity, it is notable that 2.5% ethanol diets had only a small decrease in egg laying (Figure S7) and fecundity (Figure 3) despite a 60% reduction in caloric intake (Figure S11) and no difference in weight (Table S2). This result is significant because it underscores the change in fly physiology that occurs on a 2.5% ethanol diet. In particular, it indicates that ethanol makes flies more efficient at egg production, an avenue that merits future study. The lack of a comprehensive life history tradeoff is consistent with other studies where such tradeoffs are indirect (Gould et al., 2018).

With respect to changes in gut bacteria composition on increased ethanol diets (Figure 5), reduced feeding would reduce bacterial ingestion from the food, which is a known source of the gut bacterial abundance (Blum et al., 2013). Bacteria not ingested may perish on the food because of ethanol toxicity, which would be expected to be most severe for *Acetobacter* (Figure 5E), consistent with the 10-fold reduction of *Acetobacter* observed in flies on a 2.5% ethanol diet (Figure 5B). The increased survival of bacteria in the gut may then be due both to a reduced concentration of ethanol in the fly versus the food, a bacterial strain’s ability to persist in the gut, and the ability to survive on the food until reingestion. The increased relative abundance of *L. brevis* in the gut of flies eating a high ethanol diet is thus striking, and future studies will address the mechanism of persistence.

Given how sensitive fly fitness is to dietary conditions and consumption rates (Fanson et al., 2009; Ja et al., 2009; Lee et al., 2008; Skorupa et al., 2008), the lower caloric intake of flies on ethanol diets is undoubtedly important for the various physiological factors we examined, including intestinal barrier failure (Figure 6A), intestinal cell division (Figure 6B), triacylglycerol content (Figures S9C and S9D), and many of the transcriptional responses (Table 1). In particular, reduced feeding could affect intestinal stem cell proliferation through insulin signaling (Kwon et al., 2015; O’Brien et al., 2011); the relationship to increased barrier integrity (Rera et al., 2012) is unclear and could be an interesting future direction. Likewise, the increased fat content of ethanol-fed flies is striking. Given that starvation decreases triacylglycerol content (Heier and Kühnlein, 2018), it would seem to suggest that the ethanol-fed flies are not starving, despite reduced food consumption (Figure S11) and egg production (Figure S7). This observation can only be made if we measure food consumption. Thus, it is important to account for fly feeding behavior on ethanol diets.

### Conclusion

Overall, our results suggest that low level ethanol ingestion modulates fly physiology in a manner that decreases lifespan despite caloric restriction. Flies consuming ethanol also are more efficient at converting their calories to egg production (cf. Figures 3 and S7), as based on lower feeding rates (Figure S11) and equal weights (Table S2). Interestingly, these flies also show decreased intestinal barrier permeability (Figures 6A and S9A). Our finding that the Imd pathway is implicated in this physiological shift aligns with a wealth of studies showing it is the primary pathway involved in intestinal barrier immunity (Buchon et al., 2013). While flies naturally consume low-level ethanol in the wild, laboratory diets do not explicitly include it, though live yeast may provide minor ethanol, suggesting this missing ingredient may provide new insights into fly intestinal physiology. Thus, the present work sets up future studies that focus on the mechanisms by which low level ethanol influences fly physiology.

### Limitations of the study

A primary limitation of this study is that fly genetics were not used to explicitly link the effect of specific candidate genes we identified to physiological and fitness responses. Genetics are a strength of the *Drosophila* model system, and the present work establishes a baseline for our future work that will use fly genetics to explore the cellular mechanisms of ethanol by microbiome interactions in mutant flies.

Another limitation of the present work is that we did not use gnotobiotic flies in our experiments to define the roles of individual bacterial strains. Thus, we cannot definitively attribute the fitness or phenotypic responses to a specific bacterial taxon, although *L. brevis* is the likely candidate. An advantage of fruit flies is the ability to colonize bacteria-free flies with a defined set of bacteria, e.g., (Gould et al., 2018). Our future work will build on the model of chronic ethanol ingestion that we developed here to establish causality of specific bacterial strains in the fly-microbiome-ethanol system.

The conclusions may also be qualified because of the low number of biological replicates for certain individual experiments, particularly for the range in ethanol concentration (Figure 4). Although we used mixed batches of flies and multiple experimental replicates, some assays were analyzed on a single day to reduce batch effects, including the egg-laying (Figure S7), triglyceride composition (Figures S9C and S9D), and feeding assays (Figure S11). However, somewhat mitigating this concern, we note that the conclusions are supported by multiple different experiments, many replicates within each experiment, and consistent trends that correlate with the ethanol concentration.

A final improvement to future studies will be the consistent focus on low level ethanol exposure, namely 0%, 2.5%, and 5%. These doses are in the natural exposure range for wild flies, and doses in this range elicited physiological responses in lifespan, fecundity, intestinal barrier integrity, and bacterial abundances. Applying fly genetics and gnotobiotic treatments to these concentrations will allow future work to focus on the mechanisms of the effects observed in the present work.

## Supporting citation

The following reference appears in the supplemental information: Armitage et al., 2014.

## STAR★Methods

### Key resources table

**Table:** 

REAGENT or RESOURCE	SOURCE	IDENTIFIER
**Antibodies**		

rabbit antiphospho-Histone H3 Ser10	EMD Millipore	RRID: Cat. No. 06-570

**Chemicals, peptides, and recombinant proteins**		

Erioglaucine FD&C Blue No. 1	Chem-Impex International	Cat# 22876; CAS:3844-45-9
VECTASHIELD with DAPI	Vector labs	H-1200

**Critical commercial assays**		

Free glycerol reagent	Sigma	F6428
Serum Triglyceride Determination Kit (TR0100)	Sigma	T2449
Glycerol standard solution	Sigma	G7793
Ethanol assay kit	Sigma-Aldrich	MAK076

**Experimental models: Organisms/strains**		

*Drosophila melanogaster CantonS*	Bloomington Drosophila Stock Center	BDSC 64349

**Oligonucleotides**		

See Table S4		

**Software and algorithms**		

RStudio	rstudio.com	v1.0.143, Boston, MA

### Resource availability

#### Lead contact

• Please contact Will Ludington (will.ludington@gmail.com) with any requests for further information or resources presented in this study.

#### Materials availability

• This study did not generate unique reagents.

### Experimental model and subject details

#### Fly stocks, husbandry, and creation of ethanol media

All experiments used *Wolbachia*–free *D. melanogaster* Canton-S strain (Bloomington Line 64349) as previously described (Obadia et al., 2017). Flies were maintained at 25°C with 60% humidity and 12-h light/dark cycles on autoclaved glucose-yeast medium (10% glucose, 5% Red Star brand active dry yeast, 1.2% agar, 0.42% propionic acid). Flies were three to six days old before bacterial or ethanol treatments were applied (i.e. all flies were bacteria-free and raised on 0% ethanol diets at birth). Bacteria-free flies were generated by sterilizing dechorionated embryos (Ridley et al., 2013). Bacteria-free stocks were kept for several generations before use, and they were checked regularly for presence of yeasts, bacteria, and known viruses (Gould et al., 2018). This was done by swabbing the spent vials with a sterile cotton swab after flies were transferred to fresh vials and streaking onto MRS plates. Additionally, the bacteria-free fly stocks were checked monthly for *Wolbachia* and other contaminants that might not grow on MRS by DNA extraction from flies using harsh mechanical lysis (0.1 mm zirconium beads and bead beating), which breaks open bacterial spores. Experimental vials were checked for contamination (in the case of bacteria-free treatments) and successful colonization (in the case of bacterially-colonized treatments). This was done twice during an experiment: ∼1 week after the start of the experiment and also at the experiment’s conclusion. In all cases, the swabs from bacteria-free treatments were negative and the swabs from bacterially-colonized treatments led to colonies with either *Acetobacter* or *Lactobacillus* morphology.

Bacterially-colonized flies were created by allowing approximately 50 normally-colonized young adults (from unmanipulated lab stocks) to seed autoclaved media with their frass for about 10 min, removing these flies, and then introducing bacteria-free flies. 0%–15% ethanol media was made by adding 100% ethanol to autoclaved glucose-yeast medium after it had cooled to 50°C. Vials were stored under equivalent ethanol vapor pressure to reduce evaporation until use. Because the caloric value of ethanol is not easily comparable to that of sugars (Xu et al., 2012), we did not adjust amount of glucose in an attempt to create an isocaloric diet, except where indicated, e.g. Figure S5B. Flies were transferred to fresh media every three to four days, except for the experiment that controlled for ethanol evaporation by transferring every day (Figure S5B).

### Method details

#### Ethanol concentrations of fly diets

We used a clinical grade breathalyzer to measure ethanol vapor within the headspace of a vial and use this a proxy for dietary ethanol concentration, following (Morton et al., 2014). Briefly, a 14-gauge blunt needle attached to 50 mL syringe was used to sample the headspace of vial. The sampled air was then pushed through the mouthpiece of an Intoximeters Alco-Sensor® III breathalyzer. Using 2.5%, 5%, and 10% ethanol media, with either 20 bacterially-colonized or bacteria-free flies, we checked ethanol concentration once per day for four days. Four (2.5% and 5%) or five (10%) replicate vials of each of the ethanol treatments were used. We note that preliminary experiments indicated that ethanol vapor concentration in the headspace stabilized within two hours of opening a vial or taking a measurement (data not shown).

#### Inebriation assay

Inebriation was measured using an established method (Sandhu et al., 2015). Briefly, vials were gently tapped and the number of individuals that were able to stand up 30 s later was recorded. Inebriation was measured on bacterially-colonized and bacteria-free flies on diets containing 5%, 10%, 12.5% and 15% ethanol, with four independent replicates per ethanol and bacterial treatment. As a positive control, one mL of 85% ethanol was added to a cellulose acetate plug that was pushed into the middle of a vial, and this vial was capped tightly with a rubber stopper. Within 30 min, this method led to inebriation in approximately 50% of flies under a variety of experimental conditions (Sandhu et al., 2015). To measure ethanol vapor in the positive control, a valve was attached to the rubber stopper and the headspace was sampled at 30 min using the breathalyzer method described above.

#### Internal ethanol concentration of flies

We measured the internal ethanol concentration of flies using a colorimetric enzymatic assay (Sigma-Aldrich MAK076). This approach measures the combined effects of ethanol uptake and internal metabolism. We measured ethanol concentration in individual flies fed 0% or 10% ethanol diets for 15 days. As a positive control, groups of flies not previously exposed to ethanol were enclosed in a rubber-stoppered vial with a cotton ball soaked with two mL of 35% ethanol, similar to (Fry, 2014). A dry cotton ball was added above the ethanol soaked one so that flies were unable to ingest ethanol, while still being exposed to ethanol vapor. After 60 min, individuals that could not stand, but still showed leg movements were selected. To calculate final internal concentration per fly, ethanol was considered to be primarily located in the hemolymph and the hemolymph volume was assumed to be 85 *μ*L per fly, as in (Cowmeadow et al., 2005; Troutwine et al., 2016).

#### Measurement of fecundity and lifespan

Lifespan and fecundity were measured simultaneously during the same experiment. Four replicate vials of 20 females each were created for the two bacterial treatments (bacterially-colonized and bacteria-free) and the seven ethanol treatments (0%–15%, in 2.5% increments) resulting in 56 total vials for the 14 treatments. Survival was checked each day and dead flies were removed with each transfer. Fecundity was calculated as the number of adults that emerge per transfer to new diet, divided by the number of females alive at the start of that transfer, summed over the entire experiment. Approximately 90% of all pupae that formed survived to adulthood with no differences in eclosion rate between ethanol or microbial treatments (Figure S6B) and thus only adult emergence data is shown. Development rate was measured as the day the first pupae formed following a transfer to a new vial. In a follow-up fecundity experiment that controlled for ethanol evaporation, flies were transferred to new freshly-inoculated media every day (Figure S5B). This experiment also included a diet that was isocaloric with the 2.5% ethanol diet. The isocaloric diet was created by the addition of 4.4% glucose (to the 10% glucose added to all diets) and assumes ethanol is 7 kcal/g and glucose is 4 kcal/g (Ja et al., 2007).

#### Egg laying data

A standard egg laying assay was performed (Drummond-Barbosa and Spradling, 2001).

Two replicate vials of 12 females each were created for the two bacterial treatments (bacterially-colonized and bacteria-free) and the three ethanol treatments (0%, 2.5% and 5%). A total of 72 female flies were used for this assay. After exposure to experimental conditions each fly was anesthetized with CO2 and placed into an individual molasses-attached large fly vial. Flies were allowed to lay eggs over a single 24 h period after which they were removed and eggs were counted manually via stereomicroscope.

#### Bacterial abundance within flies

This experiment was set up identical to the lifespan and fecundity experiment, except that only three replicate vials were used. On days 14, 21, 28, and 31, one to three individual flies from each replication and treatment were externally sterilized, homogenized, serially diluted, and plated onto MRS media (Obadia et al., 2017). For the 12.5% and 15% ethanol treatments, we did not sample flies on days 31, and 28 and 31, respectively, because of fly death before the end of the experiment. Eight to 16 individuals were plated per ethanol treatment (mean = 11.5). Colony forming units (CFUs) were identified by visual comparison to laboratory stocks of various species of *Acetobacter* and *Lactobacillus*, which are distinctive (Gould et al., 2018). Additionally, the identity of representative CFUs was confirmed using 16S rRNA sequencing (Data S3). While every colony with *Acetobacter* morphology that was tested was identified as *A. pasteurianus* by 16S, we cannot rule out that other *Acetobacter* species exist within this experiment at low relative abundance to *A. pasteurianus*. In only one of 81 individual flies sampled was there a CFU that had neither *Acetobacter* nor *Lactobacillus* morphology. Because this CFU morphology represented less than 2% of the total bacterial community of this fly, it was discarded as potential contamination. In all other experiments, bacterial abundance within flies was not measured. However, spent vials (i.e. after flies were transferred to fresh vials) were swabbed periodically during and after the experiment, streaked on MRS media, and the colony morphologies were noted to ensure that all experiments contained both *Acetobacter* and *Lactobacillus* and that no experiments contained any other colony morphologies.

#### Bacterial sensitivity to ethanol *in vitro*

Isolates of *A. pasteurianus*, *L. plantarum*, and *L. brevis* were grown overnight at 30°C in an appropriate medium (MYPL for *A. pasteurianus* and MRS for *L. plantarum* and *L. brevis*) and diluted to a working OD of 0.01. For *A. pasteurianus* and *L. plantarum*, growth was measured in 0%–15% ethanol media in a 96-well plate using a TECAN Infinite F200 PRO, set to 30°C and 5 min of orbital shaking per 10 min. For *L. brevis*, which forms a pellet when grown in a 96-well plate, two mL of 0%–15% ethanol MRS was inoculated with the overnight culture and shaken continuously in cell culture tubes at 30°C. After 24 h, maximum final OD was determined for each isolate, and a two-parameter Weibull function was fit to the normalized maximum ODs from the aggregate data for each strain (R package drc: Analysis of Dose-Response Curves). The inhibitory concentration for 50% growth (IC50) was calculated as the ethanol percentage that reduced normalized maximum OD by half.

#### Bacterial abundance on the diet

Experiments were set up as above, except only 0%, 7.5%, 10%, 12.5%, and 15% ethanol diets were used. On day three five flies from each of four replicates per treatment were individually homogenized, serially diluted in a 96-well plate, and pinned on selective media (MRS for *L. plantarum*, MRS + X-Gal for *L. brevis*, and MYPL for *A. pasteurianus*) using a 96-pin replicator (Boekel), (28). After fly removal from the vials, one mL of PBS and approximately ten glass beads were added. This was shaken gently on a Nutator at speed 3 for 10 min, at which time 200 *μ*L was serially diluted and pinned as above. To convert pinned colony growth to actual bacterial abundance, overnight cultures of *L. plantarum*, *L. brevis*, and *A. pasteurianus* were serially diluted in 96-well plates as above. These serial dilutions were both plated onto agar plates (to determine actual abundance) and pinned (to determine pinning efficacy). A standard curve was created relating actual abundance to growth due to pinning.

#### Intestinal barrier failure

We measured the level of intestinal barrier failure (IBF) by supplementing fly diet with 2.5% (w/v) FD&C Blue No. 1 (Rera et al., 2012). Two independent experiments were done, the first with 0% and 5% ethanol diets and the second 0%, 5%, and 7.5% ethanol diets, each with bacterially-colonized and bacteria-free treatments. For each, three or four vials of 10 flies were monitored over their entire lifespan and degree of IBF determined by the amount of blue coloration in tissues upon death. For statistical purposes, individuals in IBF categories 0 and 1 were considered IBF negative and individuals with IBF categories 2 and 3 were considered IBF positive (Clark et al., 2015). No significant differences were found between experiment 1 and experiment 2, so they were combined into a single dataset. Because the blue dye accumulates in flies with IBF and increases mortality (Clark et al., 2015), we did not directly compare the lifespan data from these IBF experiments with experiments lacking blue dye.

#### Lipid content

Bacterially-colonized or bacteria-free flies were reared on 0%, 5%, and 10% ethanol diets for 16 days, as described above. Four to ten individuals were pooled by sex (mean = 9.5), with three to five replicates for each bacteria-ethanol-sex treatment. The mass of pooled flies was determined to the nearest 1/10 of a milligram on a Mettler Toledo microbalance. Free and total lipid content was determined using established colorimetric methods (SIGMA F6428, T2449, and G7793), (Tennessen et al., 2014; Wong et al., 2014).

#### Measurement of intestinal cell division

Intestinal cell division was measured following established immunohistochemisty protocols (Guo et al., 2014). Dissected intestines were fixed at room temperature for 45 min in 100 mM glutamic acid, 25 mM KCl, 20 mM MgSO_4_, 4 mM Sodium Phosphate, 1 mM MgCl_2_ and fresh 4% formaldehyde. All washes and antibody incubations were done in PBS, 0.5% BSA, 0.1% Triton X-100. Rabbit anti-PH3 antibody was used to identify mitotic cells (kindly provided by the lab of Heinrich Jasper). Samples were mounted using VECTASHIELD® with DAPI (Vector Laboratories). The person counting mitotic cells was blinded to the identity of the samples. For experiments testing intestinal regeneration, flies were provided a filter paper soaked with 300 ul of *E. carotovora carotovora 15* (Ecc15) at OD = 100 in 5% sucrose approximately 16 h prior to dissection. Control flies were provided filter paper with 300 ul of 5% sucrose.

#### Measurement of gene expression

We used NanoStrings profiling to quantify *D. melanogaster* gene expression changes due to ethanol ingestion and bacterial colonization (NanoStrings Technologies, Inc. Seattle, WA, USA). A custom NanoStrings probeset was designed to target genes related to ethanol metabolism, innate immunity and inflammation, ethanol-mediated behavior, among others (a full list of genes, raw counts, normalized counts, and p-values are found in Data S2). Additionally, probes were designed to the bacterial 16S ribosomal RNA and alcohol dehydrogenase A and B genes. Bacterially-colonized or bacteria-free flies were reared on 0% and 10% ethanol diets for 11 days. Total RNA was obtained from individual whole flies using a Trizol extraction, following (Elya et al., 2016). 50 to 75 ng of purified RNA per sample was hybridized to the NanoString reporter and capture probesets following manufacturing instructions, and profiled on an nCounter SPRINT machine (thanks to the Laboratory of Greg Barton, UC Berkeley). Raw counts were normalized to internal NanoStrings positive and negative control probes and three housekeeping genes (Actin 5C, Gadph, and Ribosomal Protein L32). The correlation between each of the three housekeeping genes and the final normalization factor was always greater than 0.89. For all bacterial 16S genes, the normalized counts were greater than 10-fold higher in the bacterially-colonized treatments compared to the bacteria-free treatments (Data S2). For all bacterial ADH genes, the normalized counts in the bacteria-free treatment were within three standard deviations of the negative control probes and were greater in the bacterially-colonized treatments (Data S2).

RT-qPCR was performed for 0 and 10% ethanol treatments with and without bacteria. 3 biological replicates with 3 experimental vials were analyzed for each treatment. Six flies were pooled and homogenized per replicate vial. RNA was extracted in Trizol. Reverse transcription was performed using NEB LunaScript mastermix according to kit instructions; qPCR was performed according to BioRad iTaq kit instructions. Reactions were performed on a BioRad CFX96 Real Time System thermocycler. Relativefold expression was calculated relative to 0% ethanol bacterially-colonized fly treatment and normalized to ribosomal protein L32 (rpl32). Validated primers for the following target genes were used: drosomycin (Fellous and Lazzaro, 2011), diptericin (Romeo and Lemaitre, 2008), defensin (Parvy et al., 2019), PGRP-LA (Gendrin et al., 2013), PGRP SC1A (Garver et al., 2006), PGRP-SC1B (Garver et al., 2006), acetyl CoA synthase (Lyu et al., 2021), alcohol dehydrogenase (Kim et al., 2020), aldehyde dehydrogenase (Elgart et al., 2016), ribosomal protein L32 (Parvy et al., 2019).

#### Quantifying feeding behavior

In order to account for the intake rate of food under experimental conditions we measured food consumption in bacterially-colonized and bacteria-free flies on diets of 0%, 2.5% and 5% ethanol using 32P-labeled dCTP incorporation (Deshpande et al., 2014). 20 experimental female flies were allotted per vial, treatment and condition, for a total of 120 flies per time course. A stock of 20uCi/mL aqueous 32P was created and 50 μL was aliquoted into each fly food treatment and allowed to diffuse into the food over a 24 h period before use. Flies were anesthetized, placed in the irradiated fly food, and allowed to feed over the course of 24, 48, or 72 h. At the end of the time course period, vials were placed in −20°C for sample collection. Flies were then placed in individual prefilled scintillation vials and radiation levels counted with a Tri-Carb 2810 TR Liquid Scintillation Analyzer. Normalization of radiation counts to food consumption by weight was done by concurrently analyzing a weighed piece of irradiated fly food in the same fashion as the flies.

### Quantification and statistical analysis

Statistical analyses were performed in RStudio (v1.0.143, Boston, MA, USA) running R (v3.0.1, Vienna, Austria). For comparisons between treatments within an experiment, data were analyzed using pairwise *t* tests and significance was determined using a Holm-Bonferroni multiple comparison correction. Treatment effects were determined using a two-way ANOVA or an ANCOVA, except for the bacterial community dynamics experiment, which used a multivariate ANOVA (R package vegan: Community Ecology (Oksanen et al., 2016)). For the *in vitro* ethanol resistance experiment, a two-parameter Weibull function was fit to the normalized ODs from the aggregate data for each strain (R package drc: Analysis of Dose-Response Curves (Ritz et al., 2015)). For the NanoStrings gene expression experiment, significance was determined using a 5% Benjamini-Hochberg False Discovery Rate. In no cases were outlier data removed from the analyses. For all experiments, the specific statistical test used is indicated in the figure captions or main text. In the figure panels, significance level after multiple comparison correction is indicated by asterisks as follows: ∗: p < 0.05; ∗∗: p < 0.01; ∗∗∗: p < 0.001; ∗∗∗∗: p < 0.0001.

## Data Availability

• This study did not generate standard data types that would be deposited in a repository. All data generated in this study will be shared by the lead contact upon request. • No unique code was generated in this study. All code is publicly available in R repositories as reported herein. • Any additional information required to reanalyze the data reported in this paper is available from the lead contact upon request.

## Supplemental information

Document S1. Figures S1–S11 and Tables S1–S4

Data S1. Accounting for the reduced ethanol content in bacterially-colonized treatments (Figure 1D) to determine if fecundity differences are explained by ethanol loss, related to Figure 3A

Data S2. NanoStrings gene list, probe sequences, raw count data, normalized count data, and ANOVA results, related to Table 1 and Table S3

Data S3. Consensus 16 rRNA sequences from *L. plantarum*, *L. brevis*, and *Acetobacter pasteurianus*, related to Figure 5 and Figure S8

## Footnotes

Supplemental information can be found online at https://doi.org/10.1016/j.isci.2022.104000.
