# Supplementary material for: Microbiome-by-ethanol interactions impact Drosophila melanogaster fitness, physiology, and behavior
Source: iScience. 2022 Feb 28;25(4):104000. doi: 10.1016/j.isci.2022.104000 (PMC8933687; doi:10.1016/j.isci.2022.104000)
Supplement: Document S1. Figures S1–S11 and Tables S1–S4 [file mmc1.pdf]

iScience, Volume *25*

Supplemental information

Microbiome-by-ethanol interactions impact Drosophila melanogaster fitness, physiology, and behavior

James Angus Chandler, Lina Victoria Innocent, Daniel Jonathan Martinez, Isaac Li Huang, Jane Lani Yang, Michael Bruce Eisen, and William Basil Ludington

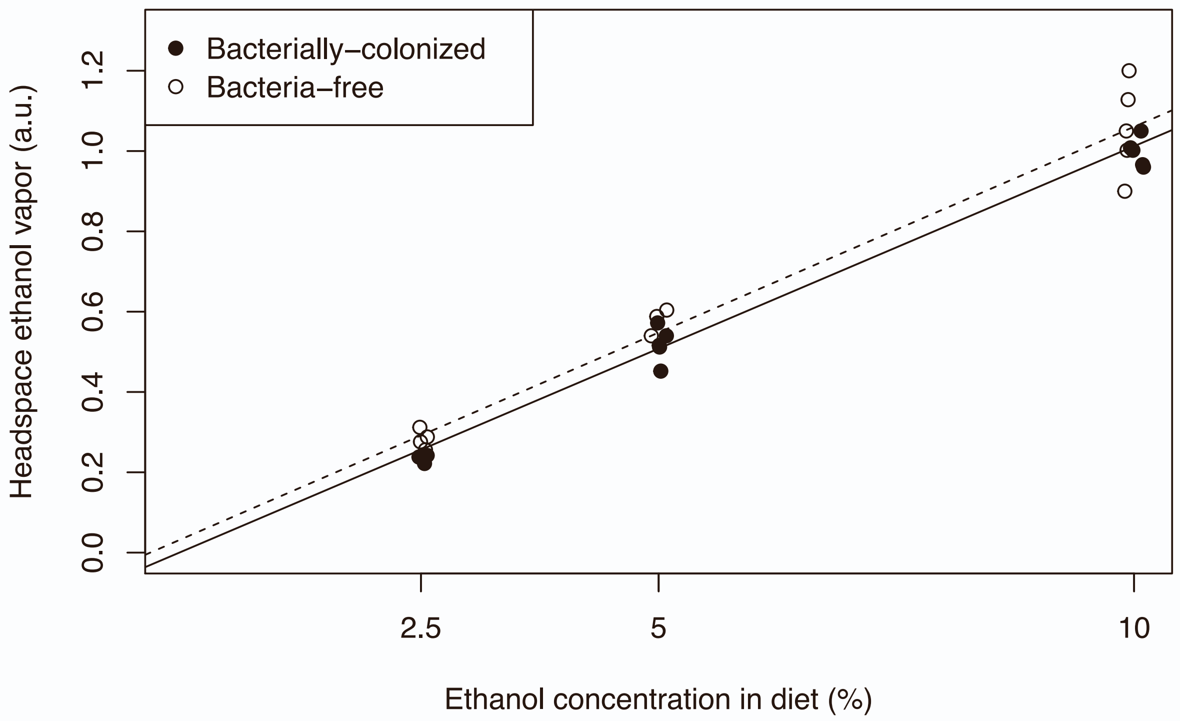

Figure S1. Vial headspace vapor is an accurate proxy for ethanol concentration in the diet, Related to Figure 1. Day zero measurements showed a strong linear correlation between headspace content and the known ethanol concentration in the media. Headspace ethanol vapor in 0% ethanol vials was between 0.001 and 0.019 (average = 0.009). Only data collected during the dietary ethanol evaporation/metabolism experiment is shown (Figure 1D to 1F).

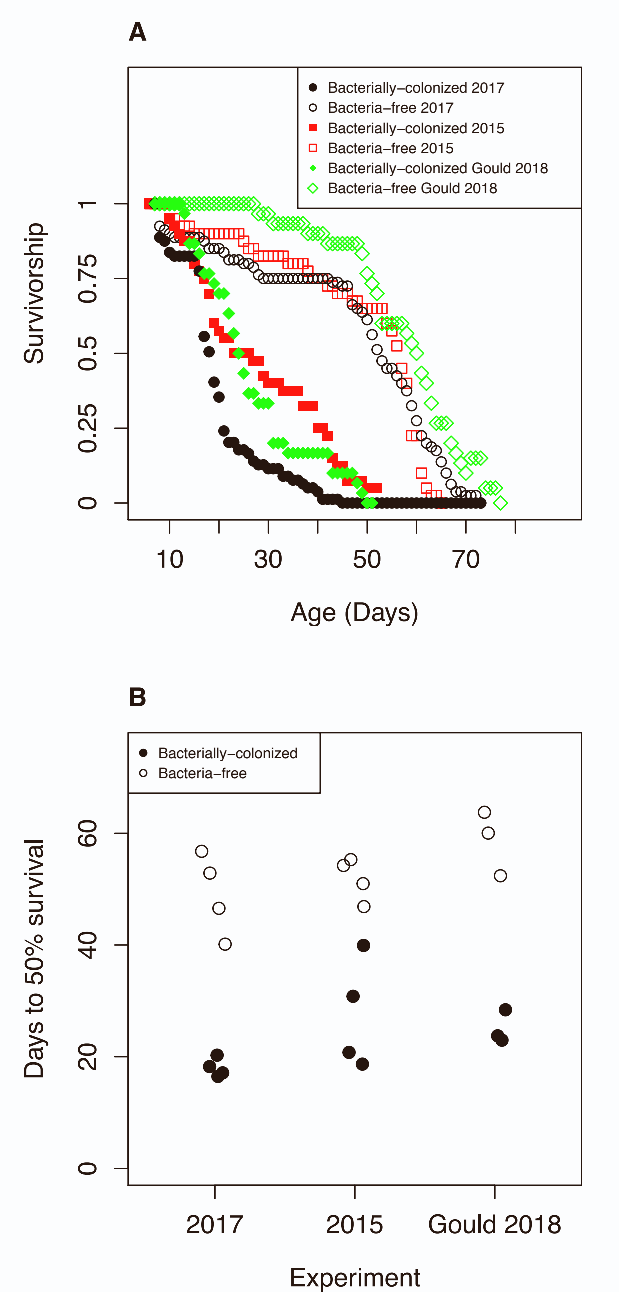

Figure S2. Decrease in lifespan for bacterially-colonized relative to bacteria-free treatments on 0% ethanol diets is consistent across experiments, Related to Figure 2. (**A**) Survival was calculated from birth and data from individual flies was pooled from either four (2017 and 2015) or three (Gould, 2018) independent replicate vials. Each treatment began with either 80 (2017), 40 (2015), or 60 (Gould, 2018) female flies. (**B**) Days to 50% survival is per replicate and calculated from birth.

For the 2017 and the 2015 data, bacterially-colonized flies were created by allowing normally-colonized young adults (from unmanipulated lab stocks) to seed autoclaved media with their frass, removing these flies, and then introducing bacteria-free flies (see methods). For the Gould et al. data, the treatment labelled as bacterially-colonized were conventionally-reared adults rather than being reared germ-free and seeded with bacteria as adults. The Gould data is part of a larger dataset. Notably, we do not find a major difference in lifespan between these different colonization methods.

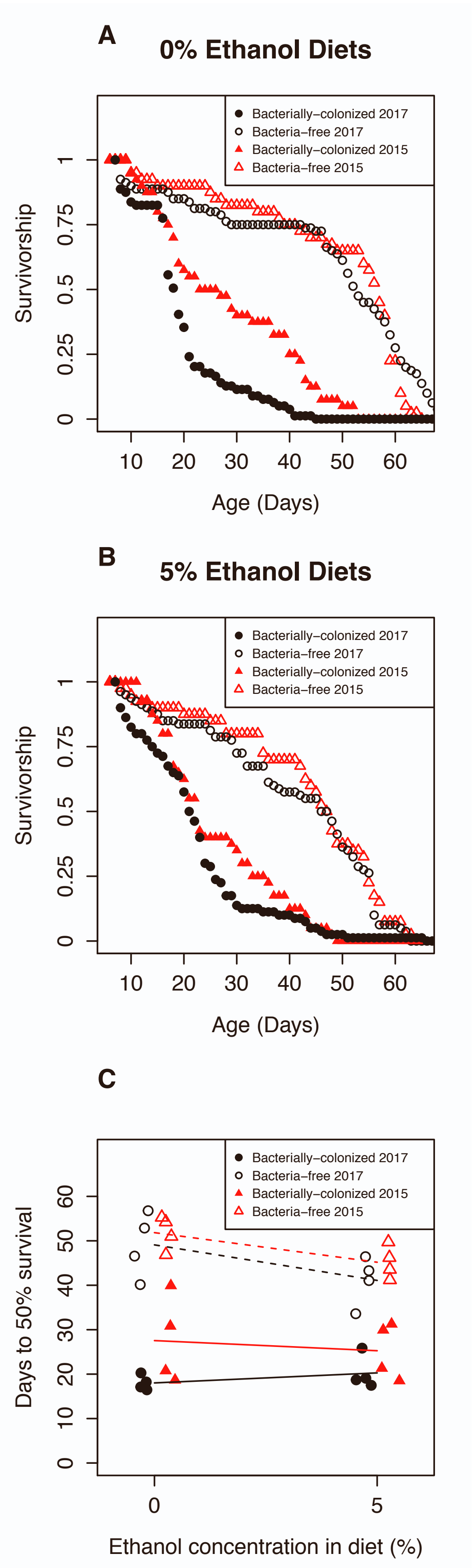

Figure S3. Effect of bacteria and ethanol on fly lifespan is consistent across experiments, Related to Figure 2. (**A** and **B**) Survival was calculated from birth (see methods) and data from individual flies was pooled from four independent replicate vials. Each treatment began with either 80 (2017) or 40 (2015) female flies. **A**: 0% Ethanol Diets. **B**: 5% Ethanol Diets. (**C**) Days to 50% survival is per replicate and calculated from birth. Each replicate vial began with either 20 (2017) or 10 (2015) female flies. Lines show the best fit trends for each treatment and date (solid black: bacterially-colonized 2017, R^2=0.16; dashed black: bacteria-free 2017, linear, R^2=0.34; solid red: bacterially-colonized 2015, R^2=0.02; dashed red: bacteria-free 2015, linear, R^2=0.51). 2017 data is from the experiment shown in Figure 2 and performed by the authors JAC, LVI, and JLY in 2017. 2015 data is from an experiment performed by JAC in 2015.

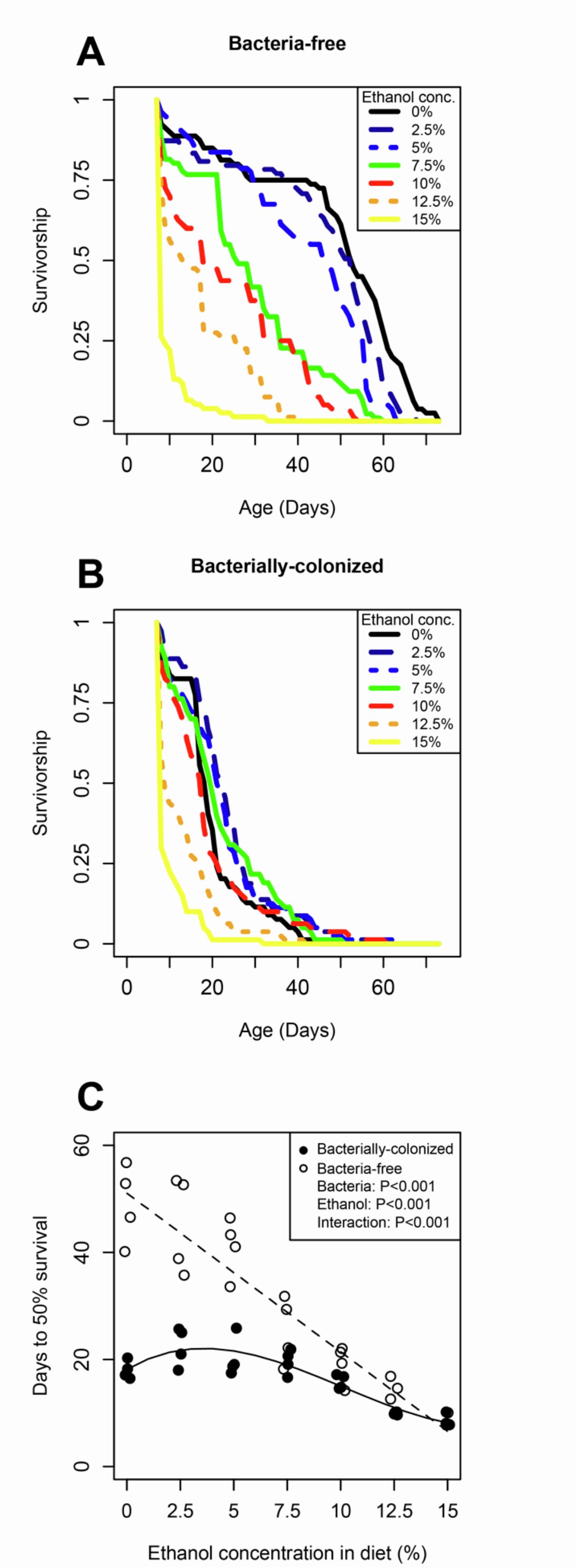

Figure S4: Bacteria mediate the effect of ethanol on fly lifespan, Related to Figure 2. Survivorship (A and B) and days to 50% survival (C) of (A) bacterially-colonized and (B) bacteria-free flies fed diets containing 0% to 15% ethanol in 2.5% increments. Bacterial colonization and ethanol reduced fly lifespan, with the negative effect of ethanol greater in magnitude and more significant in bacteria-free flies. Results from replicate experiments with 0% and 5% ethanol diets is shown in Figures 3 and S3. Survival is time from adult eclosion from the pupal case (see methods) and data from individual flies was pooled from four independent replicate vials. Each treatment began with 4 vials of 20 flies each. (C) A Robust 2-way ANOVA (R package WSR2) finds bacteria, ethanol, and the bacteria*ethanol interaction to be significant at $p<0.001$. Days to 50% survival is per replicate vial. Lines show the best fit trends for each bacterial treatment (Solid: bacterially-colonized, third order, $R^2=0.83$; Dashed: bacteria-free, linear, $R^2=0.87$).

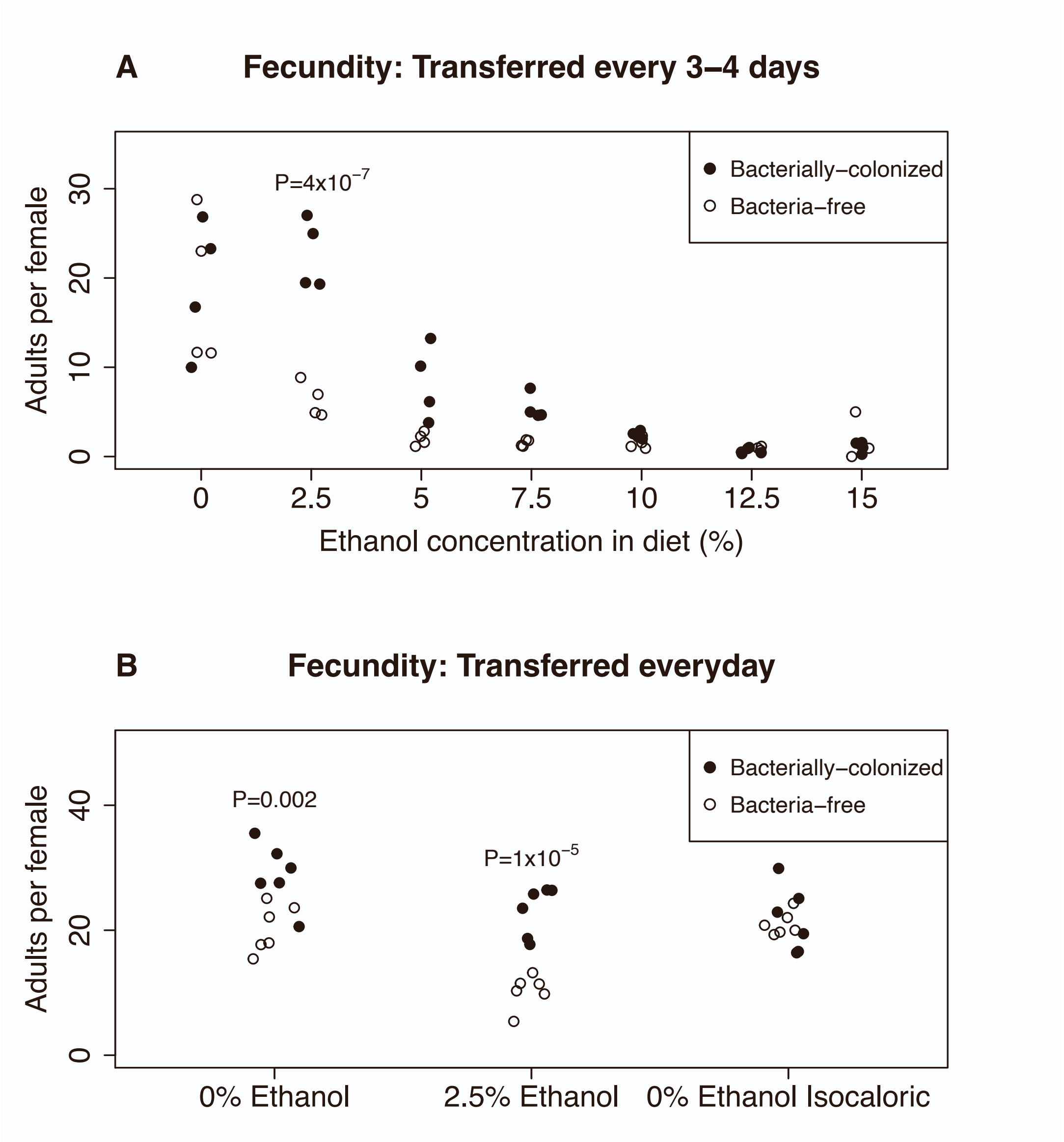

Figure S5: Bacteria mediate the effect of ethanol on fly fecundity, Related to Figure 3. Adult progeny per female was calculated as the average total number of progeny per female over the entire lifetime (see methods). Each replicate began with either **(A)** 20 or **(B)** 10 females. p-values are calculated from a pairwise *t* test between bacterial treatments for a given ethanol concentration and are Holm-Bonferroni corrected for multiple comparisons. Non-significant p-values are not shown. **(A)** Bacteria reduced the negative effects of ethanol on fly fecundity at 2.5% ethanol. Flies were transferred to new diets every 3-4 days. N=4 experimental replicates per treatment. **(B)** Increased fecundity on a 2.5% ethanol diet occurred with daily transfer to fresh ethanol food and was not due to the caloric contribution of ethanol. N=6 experimental replicates per treatment.

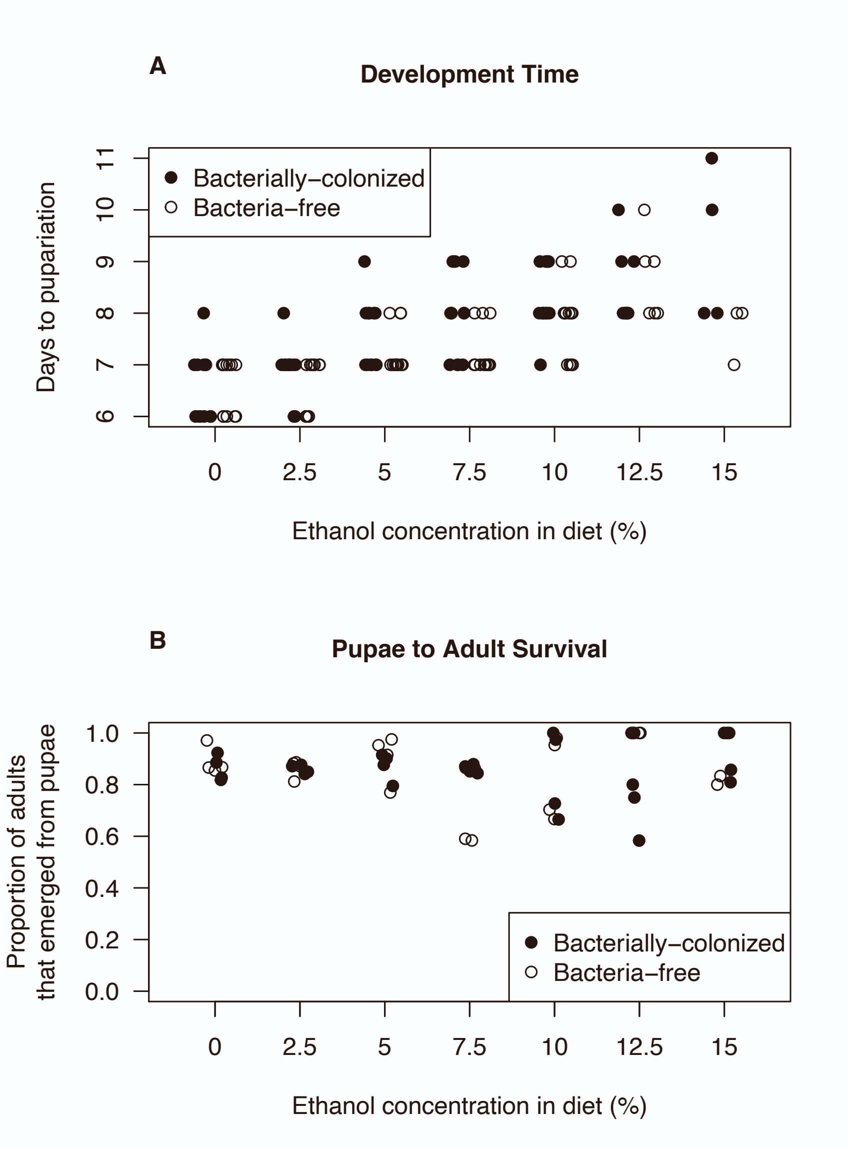

Figure S6. No effect of bacterial colonization on larval development time and pupa to adult survival, Related to Figure 3. (**A**) Bacterial colonization did not affect development time. Each point represents the day that the first pupal case was observed. Days were measured as the time since flies were first transferred into the vial. Because egg-laying dropped in later life, data shown is for the first three transfers (age 18 days). Note no difference between 0% and 2.5% ethanol treatments, suggesting ethanol did not affect larval development at this concentration. (**B**) Survival during metamorphosis was not affected by ethanol or bacterial colonization. The increasing variance at higher ethanol concentrations can be attributed to sampling error due to low absolute fecundity rate on ethanol diets greater than 5%. All data were collected during the experiment in Figure 4.

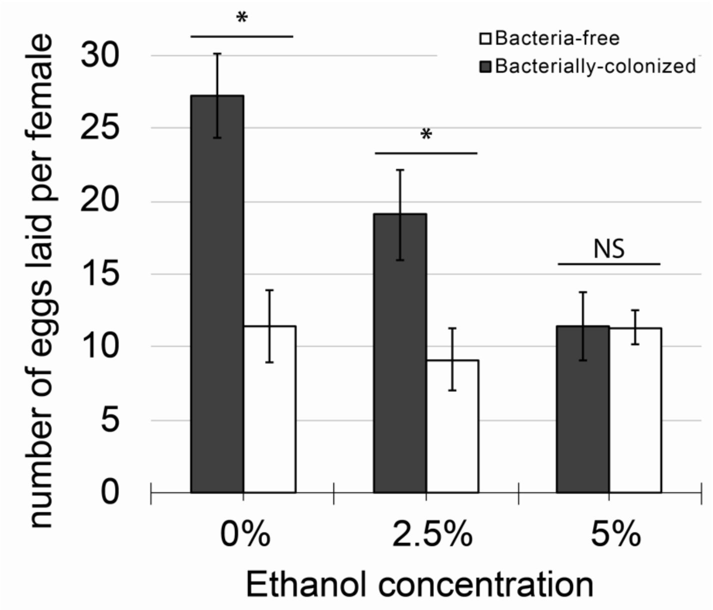

Figure S7: Bacteria-free flies lay fewer eggs relative to bacterially-colonized flies, Related to Figure 3. For each treatment 12 total flies from 3 independent stocks of flies reared on the corresponding ethanol concentration were tested individually (72 flies total). Flies were taken from their ethanol treatment and put on molasses agar with wet yeast paste. Eggs were counted after 24 h of laying (see STAR Methods). Data represented as mean +/- SEM. Fecundity compared between flies +/- bacteria for each ethanol concentration. NS: not significant. *: $p<0.05$. One-sided t test at each ethanol concentration to test whether lack of bacteria reduces fecundity. p-values have been Bonferroni corrected for 3 comparisons.

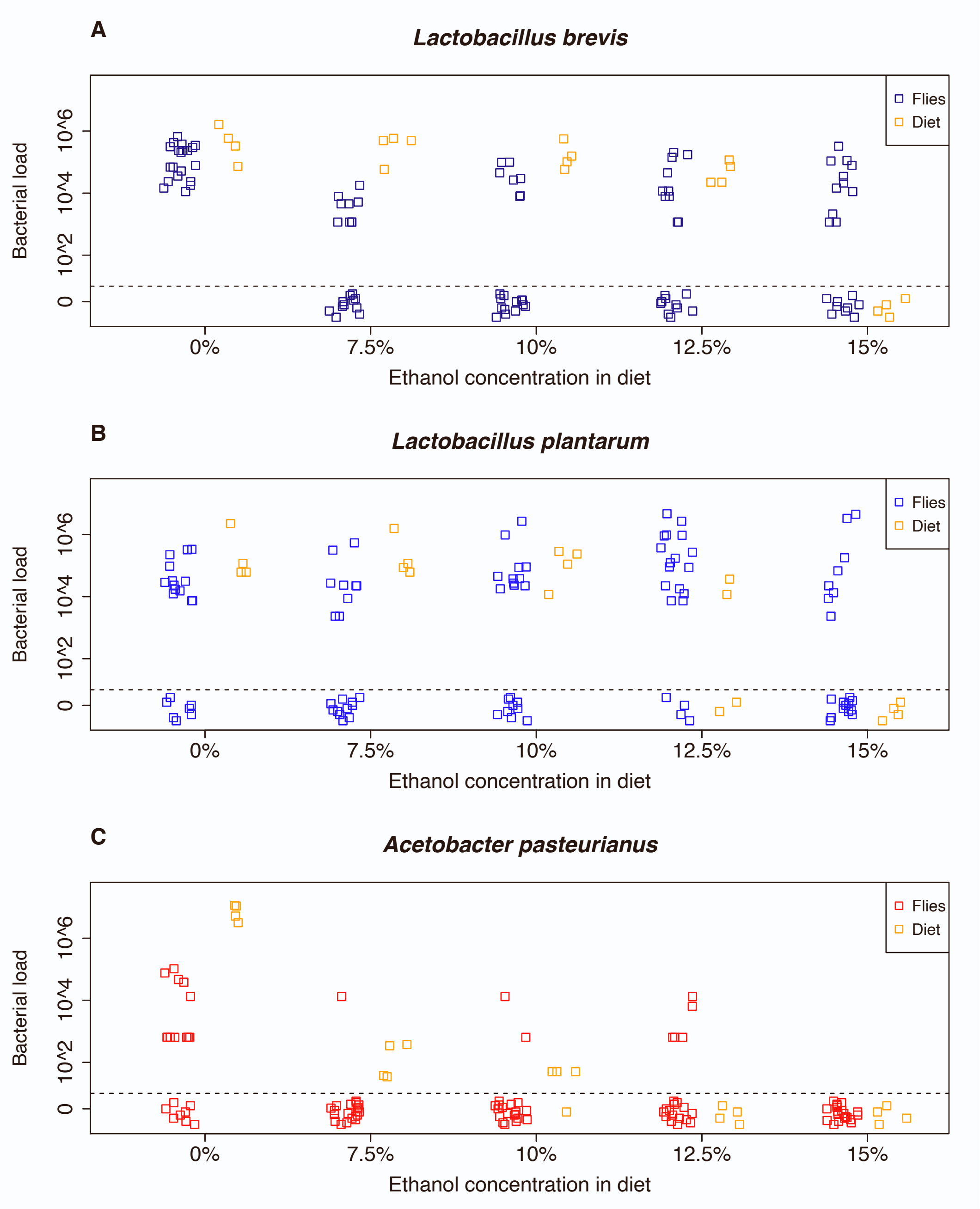

Figure S8. Bacteria are present within flies even when undetectable on their food, Related to Figure 5. Bacterial load was determined in homogenized flies and the surface of their diet. Data are presented separately for (**A**) *L.* brevis, (**B**) *L. plantarum*, and (**C**) *A. pasteurianus*. Each dark blue/blue/red point represents an individual fly (five per replicate, 20 total per ethanol concentration) and each orange point represents the food from a vial (four per ethanol concentration). All points below the dashed line are 0 and are expanded for clarity. Note that *L. brevis* and *L. plantarum* were not present on 15% ethanol medium, despite high abundance

within approximately half of the flies on that diet and *A. pasteurianus* was not present on 12.5% ethanol medium, despite high abundance in five flies on that diet. Also note the same overall trend as Fig 5, with *A. pasteurianus* being more sensitive to ethanol than *L. brevis* and *L. plantarum*.

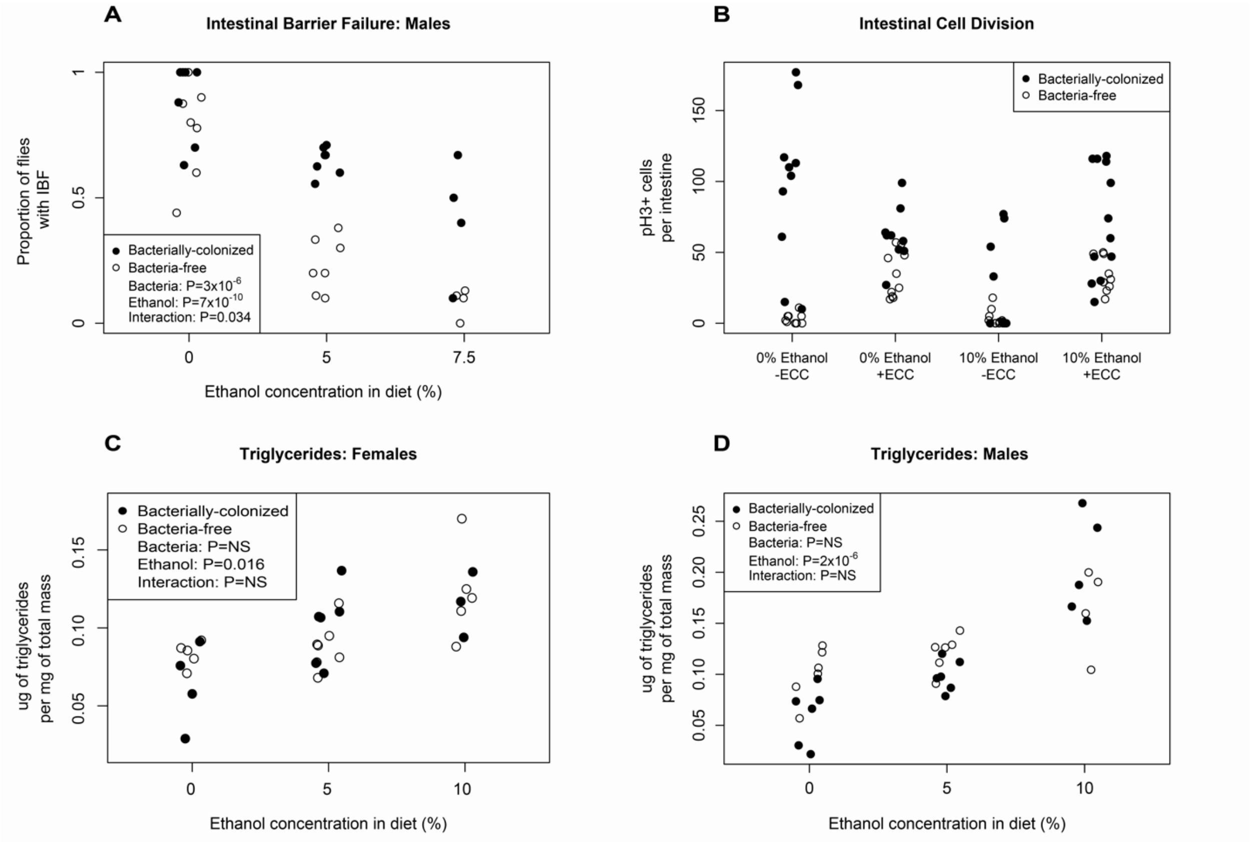

Figure S9. Bacteria mediate the effect of ethanol on fly physiology, Related to Figure 6. (**A**) The prevalence of intestinal barrier failure (IBF) in male flies decreases with dietary ethanol and this decrease is more pronounced in bacteria free flies. Each point represents the average from a replicate vial. Flies were scored within 24 hours of death. Values in the legend are the results from an ANCOVA. Female data shown in Figure 6A. (**B**) Ethanol reduces mitotic cells in the intestine in bacterially colonized but not bacteria free flies. Ethanol did not change the number of PH3+ cells during intestinal regeneration following oral ingestion of Ecc15. The Ecc15 feeding experiment was conducted on a single day with 2 to 4 independent feedings of Ecc15 for each treatment. Each data point indicates the number of PH3+ cells in an individual intestine. Values in the legend are the results from a two-way ANOVA. Comparison p-values were calculated from a pairwise *t* test between treatments and were Holm-Bonferroni corrected for multiple comparisons. (**C & D**) Ethanol ingestion increases stored triglycerides in male flies, regardless of bacterial treatment. Each point represents a pooled sample of 4 to 10 flies. Values in the legend are the results from an ANCOVA. Female data shown in Fig 6D.

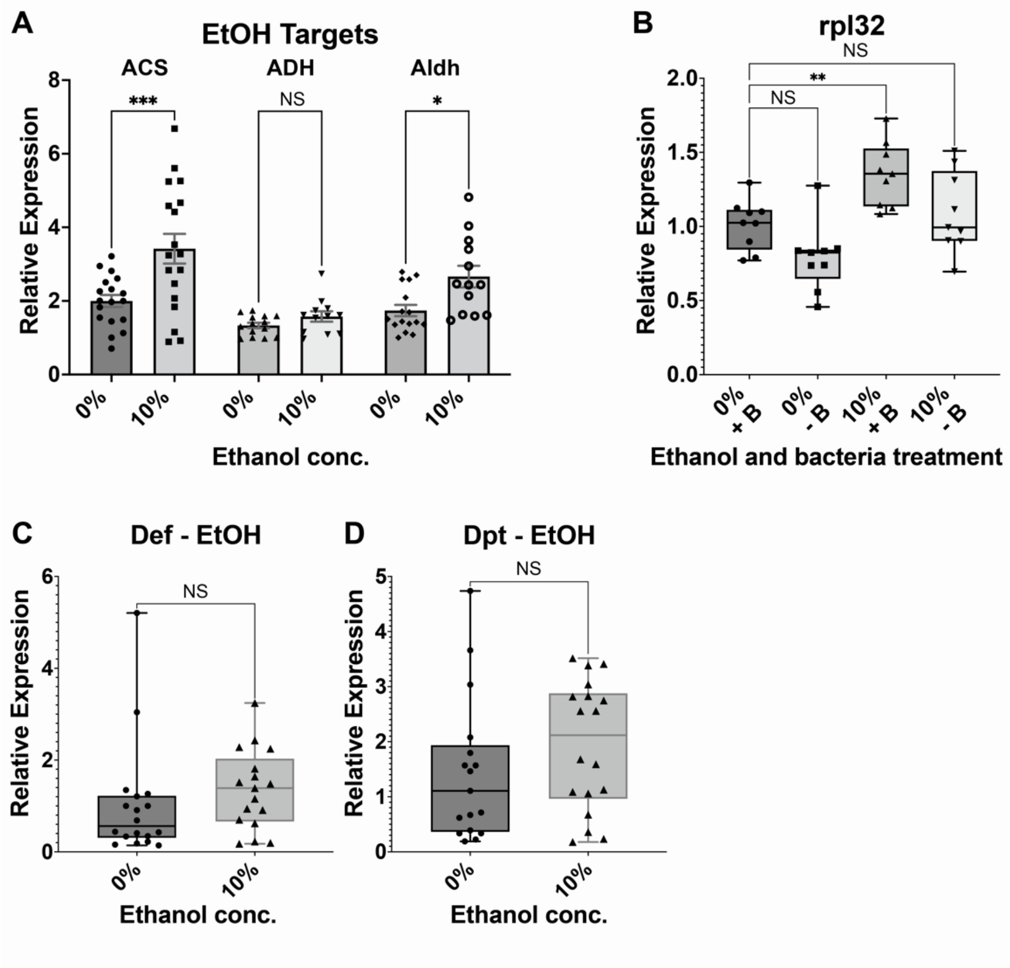

Figure S10. RT-qPCR of transcriptional response to ethanol, Related to Figure 7. (**A**) Ethanol target genes acetyl coA synthase, alcohol dehydrogenase, and aldehyde dehydrogenase. (**B**) The control gene for normalization, ribosomal protein L32. Immunity genes (**C**) defensin, and (**D**) diptericin. Methods are identical to Figure 7. Individual replicates shown. In A, data are represented as mean +/- SEM. In B-D, box represents interquartile range, horizontal bar is median, and whiskers are total data range.

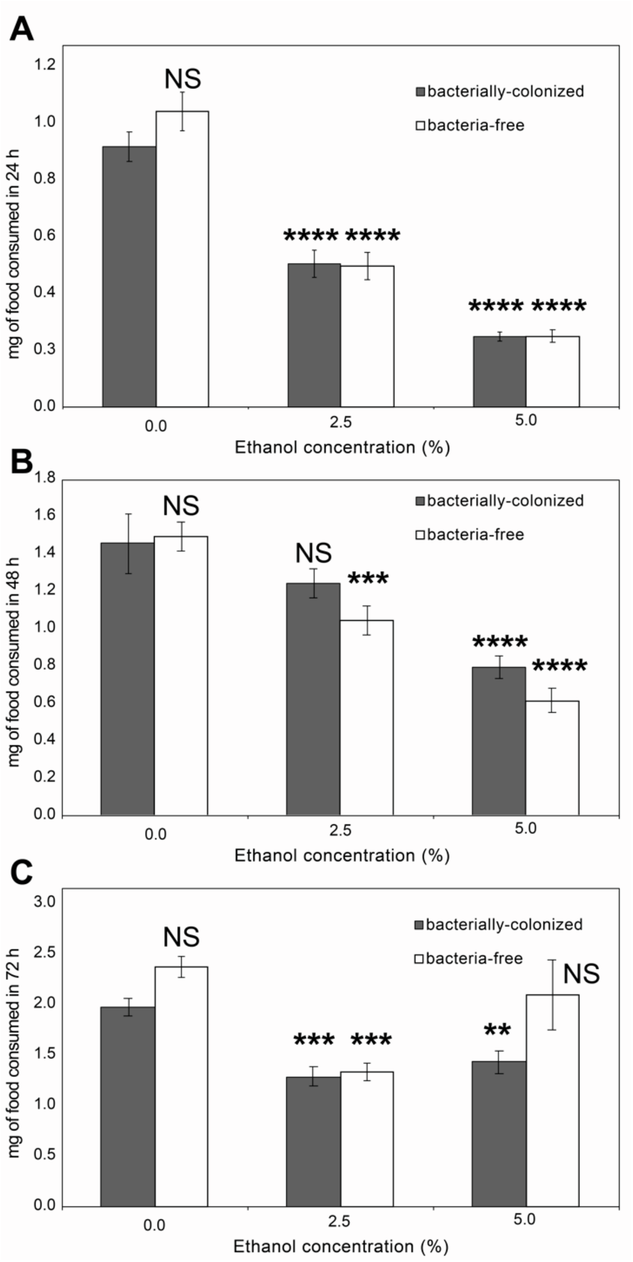

Figure S11. Flies consume less food on ethanol diets, with no effect of bacterial colonization, Related to Figures 2 and 3. Food consumption was quantified using 32P-labeled dCTP incorporation. Female flies were allowed to feed for (**A**) 24 h, (**B**) 48 h, or (**C**) 72 h. For each treatment 18 to 20 flies were individually tested (mean 116 total flies per timecourse; mean 19.3 flies per treatment). A one-sided t test, with Bonferroni correction for multiple comparisons was performed to test the hypothesis that ethanol decreases food consumption as compared to the 0% ethanol bacterially-colonized control flies. Consumption was normalized to weight of food

sampled from the same vial at time of collection. Data represented as mean +/- SEM. The reduced trend at 72 h is presumably due to evaporation of ethanol over the feeding period. NS: not significant, **: $p<0.01$, ***: $p<0.001$, ****: $p<0.0001$. No differences were detected between bacteria-free and bacteria-colonized treatments at the same ethanol concentration.

		Days to 50% Survival		
Bacterial Treatment	Ethanol Treatment	Mean	SD	SEM
Bacteria-free	0%	49	7.3	3.7
	2.5%	45	9.2	4.6
	5%	41	5.5	2.8
	7.5%	25	6.3	3.2
	10%	19	3.5	1.8
	12.5%	13	3.1	1.6
	15%	8.4	1.1	0.6
Bacterially-colonized	0%	18	1.6	0.8
	2.5%	22	3.6	1.8
	5%	20	3.7	1.9
	7.5%	20	2.3	1.2
	10%	16	1.3	0.7
	12.5%	10	0.2	0.1
	15%	8.5	1.1	0.6

Table S1. Bacterial colonization and ethanol negatively affect fly lifespan, Related to Figure 2. Days to 50% survival is per replicate. All data is calculated from birth (see STAR Methods).

	Sex	Effect of Ethanol	Effect of Bacteria	Interaction
Mass	Males	NS	NS	NS
	Females	NS	NS	NS
Free Glycerol / Mass	Males	NS	NS	NS
	Females	NS	NS	NS
Stored Triglycerides / Mass	Males	$1.8x10^{-6}$	NS	NS
	Females	0.016	NS	NS

Table S2. Ethanol ingestion increases stored triglycerides in flies, regardless of bacterial treatment, with no change in mass or free glycerides, Related to Figure 6. p-values represent the results of a 2-way ANOVA.

	Bacterially-colonized to bacteria-free fold-change			10% ethanol to 0% ethanol fold-change		
	All samples	0% ethanol only	10% ethanol only	All samples	B- only	B+ only
AttacinA/B[1]	15	30	10	0.6	1.0	0.6
AttacinC	89	70	96	1.0	2.8	0.9
AttacinD[2]	4.0	3.6	4.2	3.5	2.5	3.5
CecB	4.0	3.6	4.2	2.2	2.0	2.3
CecropinA1	69	47	80	3.5	2.1	3.6
CecropinA2	54	130	34	1.0	3.8	1.0
Defensin	5.3	6.2	4.6	0.9	1.2	0.8
DiptericinA	17	8	34	2.0	0.5	2.2
DiptericinB	59	49	70	1.2	0.8	1.2
Drosocin	59	54	63	1.2	1.1	1.2
Drosomycin	1.9	1.3	2.3	1.8	1.3	2.2
Drosomycin-like 2	4.5	8.2	2.0	0.5	1.5	0.4
Drosomycin-like 3	1.7	3.2	0.7	0.6	1.5	0.3
Drosomycin-like 4	0.7	0.9	0.4	0.6	0.8	0.4
Drosomycin-like 5	1.9	1.3	2.3	1.8	1.3	2.2
Metchnikowin	58	42	96	0.9	0.4	0.9
Average all AMPs	28	29	31	1.5	1.5	1.49
Average significantly different than 1?	Yes	Yes	Yes	Yes	Yes	No

Table S3. Bacterial colonization and dietary ethanol can separately induce the expression of anti-microbial peptides (AMPs), Related to Table 1 and Figure 7. Fold-change was calculated from normalized NanoStrings expression counts. Values greater than one indicate an increase in expression due to either bacteria (left side of table) or ethanol (right side). Significance was determined if the 99% confidence interval of the average of all AMPs was different than one (i.e. no fold-change). B+, Bacterially-colonized. B-, Bacteria-free.

[1]No probe could be designed to differentiate AttacinA and AttacinB and therefore a consensus probe (identical to both) was used.

[2]For AttacinD, the average normalized counts were within two standard deviations of the negative control probes and thus these values may not be accurate.

Oligonucleotides		
5’-GCACCCACATCTGGAACGA	(Garver et al., 2006)	PGRP-SC1A-fwd
5’-CGTGGTTTGTGTCAAGGACCTA	(Garver et al., 2006)	PGRP-SC1A-rev
5’-CGCACTCACATCTGGAACGA	(Garver et al., 2006)	PGRP-SC1B-fwd
5’-CCTTTTCAGGAACCACTCTAACCA	(Garver et al., 2006)	PGRP-SC1B-rev
5’-AGGCCCAAGATCGTGAAGAA	(Parvy et al., 2019)	rpl32-fwd
5’-TGTGCACCAGGAACTTCTTGA	(Parvy et al., 2019)	rpl32-rev
5’-CCTTTATGGGCGACTATGGC	(Gendrin et al., 2013)	PGRP-LA-fwd
5’-CTTGGCGTCCCACGATTC	(Gendrin et al., 2013)	PGRP-LA-rev
5’-CTGCCTGTCCGGAAGATACAA	(Armitage et al., 2014)	Drosomycin-fwd
5’-5’-TCCCTCCTCCTTGCACACA	(Armitage et al., 2014)	Drosomycin-rev
5’-GCTGCGCAATCGCTTCTACT	(Armitage et al., 2014)	Diptericin-fwd
5’-TGGTGGAGTGGGCTTCATG	(Armitage et al., 2014)	Diptericin-rev
5’-CTTCGTTCTCGTGGCTATCG	(Parvy et al., 2019)	Defensin-fwd
5’-ATCCTCATGCACCAGGACAT	(Parvy et al., 2019)	Defensin-rev
5’-AAATCCCATGGACCGATGAC	(Lyu et al., 2021)	Acetyl-CoA synthase-fwd
5’-TGTAGAGCATGAACAATGGATCCT	(Lyu et al., 2021)	Acetyl-CoA synthase-rev
5’-CCGGTGGTATCATCTGCAAC	(Kim et al., 2020)	Alcohol dehydrogenase-fwd
5’-CCAGGAGTTGAACGTGTGCA	(Kim et al., 2020)	Alcohol dehydrogenase-rev
5’-AGAACTTCGCAGCAGCTGTTG	(Elgart et al., 2016)	Acetaldehyde dehydrogenase-fwd
5’-TGTTGATAAATACCCCGGTGTAGA	(Elgart et al., 2016)	Acetaldehyde dehydrogenase-fwd

Table S4. Oligonucleotide primer sequences used for qPCR. Related to Figure 7 and Figure S10.
